# The Phylogeny, Biodiversity, and Ecology of the *Chloroflexi* in Activated Sludge

**DOI:** 10.3389/fmicb.2019.02015

**Published:** 2019-09-13

**Authors:** Lachlan B. M. Speirs, Daniel T. F. Rice, Steve Petrovski, Robert J. Seviour

**Affiliations:** ^1^La Trobe Institute for Molecular Sciences, La Trobe University, Bendigo, VIC, Australia; ^2^Department of Physiology, Anatomy and Microbiology, La Trobe University, Bundoora, VIC, Australia

**Keywords:** *Chloroflexi*, activated sludge, bulking, foaming, filamentous bacteria FISH, amplicon sequencing

## Abstract

It is now clear that several of the filamentous bacteria in activated sludge wastewater treatment plants globally, are members of the phylum *Chloroflexi.* They appear to be more commonly found in treatment plants designed to remove nitrogen (N) and phosphorus (P), most of which operate at long sludge ages and expose the biomass to anaerobic conditions. The *Chloroflexi* seem to play an important beneficial role in providing the filamentous scaffolding around which flocs are formed, to feed on the debris from lysed bacterial cells, to ferment carbohydrates and to degrade other complex polymeric organic compounds to low molecular weight substrates to support their growth and that of other bacterial populations. A few commonly extend beyond the floc surface, while others can align in bundles, which may facilitate interfloc bridging and hence generate a bulking sludge. Although several recent papers have examined the phylogeny and *in situ* physiology of *Chloroflexi* in activated sludge plants in Denmark, this review takes a wider look at what we now know about these filaments, especially their global distribution in activated sludge plants, and what their functional roles there might be. It also attempts to outline why such information might provide us with clues as to how their population levels may be manipulated, and the main research questions that need addressing to achieve these outcomes.

## Introduction

### The Activated Sludge Process

Treatment of domestic and industrial wastewater is essential to reduce potentially harmful levels of organic and inorganic compounds and pathogenic microbes to those allowing the treated water to be run into a receiving water body, such that its self-purification ability is not compromised (Catherine et al., 2013; Gaget et al., 2017). The most popular treatment process globally is activated sludge (Daigger, 2014), and in use now for more than 100 years (Lofrano and Brown, 2010; Jenkins and Wanner, 2014). No longer are these seen as disposal systems, but as valuable sources of recycled water, and the biomass or sludge for recovery of chemicals of value (van Loosdrecht et al., 2014; Puyol et al., 2016). This biomass consists primarily of bacteria and protozoa, which become organized as aggregates called ‘flocs.’ It is generally accepted that the *Chloroflexi*, the subject of this review, play an important role in providing the filamentous matrix around which desirable strong flocs with rapid settling properties are formed (Kragelund et al., 2007a; Wanner and Jobbagy, 2014; Nierychlo et al., 2019). Through continuous recycling, the populations best suited to treat the raw incoming sewage, are selected (Daigger, 2014; Jenkins and Wanner, 2014).

As discharge license requirements became more stringent, plant configurations evolved to reduce effluent nitrogen (N) and phosphate (P) concentrations to environmentally acceptable levels. In both, further selective pressures are imposed to encourage the growth of bacterial populations whose ecophysiology ensures N and P removal (Barnard and Comeau, 2014; Khunjar et al., 2014; Stensel and Makinia, 2014). These systems were developed largely empirically with no knowledge of the microbes responsible, but subsequently, N removal processes including the SHARON, CANON, N-Damo and ANAMMOX processes have been designed, based on detailed understanding of the bacteria involved (Schmidt et al., 2003; Kartal et al., 2013; van Kessel et al., 2018).

### Microbiology of Activated Sludge Communities

Only in the past 30 years have we begun to understand activated sludge microbiology, an outcome coinciding with development of culture independent molecular methods (Nielsen and McMahon, 2014). Development of PCR, cloning and Sanger DNA sequencing allowed compositions of activated sludge communities to be elucidated, with no need to culture individual members, being based instead on using 23S and 16S rRNA gene sequence analyses used as phylogenetic markers (Nielsen and McMahon, 2014). Subsequent design of fluorescently tagged oligonucleotide probes targeting RNA sequences of populations of interest allowed *in situ* Fluorescence *in situ* Hybridization (FISH) identification of individual cells (Amann and Fuchs, 2008; Nielsen et al., 2009a; Seviour, 2010b; Noguera et al., 2014). In combination with histochemical staining, stable isotope probing (SIP) and microautoradiography (MAR) (McIlroy et al., 2017a), FISH has elucidated the *in situ* ecophysiology and possible function/s of individual probed populations at the single cell level. Such data have revealed the true biodiversity of activated sludge communities, and presence of previously unknown populations existing there, including the *Chloroflexi* discussed in this review, where most have yet to be cultured. The impact of next generation DNA (ngs) amplicon sequencing/metagenomics approaches on our understanding of the *Chloroflexi* in activated sludge communities will be discussed later.

## Nutrient Removal Plants

### Microbiology of Plants Designed to Remove Nitrogen

Continuous flow aerobic activated sludge plants removing N have sequential reactors where the influent is passed continuously between aerobic and anoxic zones. In the former, nitrification occurs where ammonia is oxidized first to nitrite and then nitrate by the nitrifying chemolithoautotrophic *Bacteria.* It was always thought that nitrification involved two different populations, the **nitroso** bacteria responsible for ammonia oxidation to nitrite, and the **nitro** bacteria oxidizing nitrite to nitrate (Daims et al., 2015). FISH has revealed that clusters of each co-exist *in situ* in a mutualistic dependent syntrophic relationship, where the nitroso bacteria supply the nitro bacteria with their energy source nitrite, and in return the nitro bacteria remove this harmful chemical, and supply the nitroso bacteria with ammonia generated from their high urease activity (Daims et al., 2015). We know now that a single population can oxidize ammonia all the way to nitrate. These are the ‘Comammox’ *Nitrospira* related bacteria, and occur widely, including in N removal wastewater treatment plants (Daims et al., 2015; Roots et al., 2019). Equally unexpectedly, Sorokin et al. (2014) isolated successfully a non-filamentous *Chloroflexi* nitrifying bacterium, *Nitrolancea hollandica*, which this review shows occurs in activated sludge systems (see later).

Under ‘anoxic’ conditions, nitrate/nitrite acting as the terminal electron acceptor is then reduced sequentially by denitrifying chemoorganoheterotrophic bacteria using anaerobic respiration to eventually inert dinitrogen gas. In many plants, partially reduced nitrous oxide and nitric oxide are released instead (Foley et al., 2010; Massara et al., 2017). Both are highly persistent greenhouse gases responsible for global warming and climate change.

Molecular techniques have shown that previously unknown *Nitrosomonas* and diverse *Nitrospira* and *Nitrotoga* populations are the most abundant nitrifiers in activated sludge (Daims et al., 2015). Identities of the bacteria important in denitrification, leading to dinitrogen formation and N removal (Lu et al., 2014) show too that most are as yet uncultured bacterial populations (McIlroy et al., 2014).

### Microbiology of Plants Designed to Remove Phosphorus

Phosphorus removal is most efficiently achieved biologically by selectively encouraging the proliferation of polyphosphate accumulating organisms (PAO), which accumulate and store intracellular P as polyP granules. The configurations and operating conditions required to achieve this process of Enhanced Biological Phosphate Removal (EBPR) are well proven (Barnard and Comeau, 2014), and will not be detailed here. Essentially the biomass is recycled repeatedly between anaerobic (feed), and aerobic (famine) conditions. Under anaerobic conditions, readily biodegradable substrates in raw sewage are used by PAO to synthesize intracellular storage products using polyphosphate stored under aerobic conditions as main energy source. Under aerobic conditions, with no readily biodegradable substrates available, the PAO use their storage products as energy and carbon sources, and hence are selectively favored. PAO assimilate orthophosphate from the bulk liquid and store it intracellularly as polyphosphate granules (Seviour et al., 2003; Oehmen et al., 2007; Seviour and McIlroy, 2008; McMahon et al., 2010). Their subsequent removal by sludge wasting removes cellular immobilized phosphorus. All plants removing P also remove N.

Equally profound changes in PAO identification have resulted using molecular approaches. Thus, 16S rRNA gene cloning and FISH/MAR/histochemical staining showed that the betaproteobacterial *Candidatus* ‘Accumulibacter phosphatis’ was a common PAO, the first identified (Oehmen et al., 2007; McMahon et al., 2010; He and McMahon, 2011). Actinobacterial *Tetrasphaera* species are also PAO, and occupy a different niche in EBPR communities (Marques et al., 2017). Other putative PAO have been proposed, and their claims to be PAO are assessed by Stokholm-Bjerregaard et al. (2017).

The so-called glycogen accumulating bacteria GAO, are thought to share the PAO anaerobic phenotype (McIlroy et al., 2018), but instead of synthesizing polyP aerobically under aerobic famine conditions, they store glycogen (Stokholm-Bjerregaard et al., 2017; Nielsen et al., 2019). They too are phylogenetically and metabolically diverse. While some store PHA anaerobically as many PAO do, the storage products in others can vary (Stokholm-Bjerregaard et al., 2017; McIlroy et al., 2018).

## Microbiological Solids Seperation Problems

Most activated sludge plants around the world suffer intermittently from the problems of bulking and foaming (Seviour and Nielsen, 2010; Wanner and Jobbagy, 2014; Rossetti et al., 2017). Both can be caused by excessive proliferation of filamentous bacteria (Jenkins et al., 2003; Martins et al., 2004; Wanner et al., 2014). When filaments extend from the floc surface into the bulk liquid, floc sedimentation velocity is retarded. In extreme cases bulking may result. Alternatively, excessive filament growth can affect floc settlability by rendering them more diffuse (Martins et al., 2004; Burger et al., 2017). In both cases, biomass is then released with the treated liquid effluent into the environment, increasing effluent COD. Membrane reactors are becoming popular because they overcome any floc settling although filamentous bacteria may cause membrane fouling (Crawford et al., 2014).

Activated sludge foam formation is a flotation event, readily visible on the surface of the aerobic reactors. Such foams can lead to serious changes in the operation of the activated sludge process, and in some cases, serve as the source of opportunistic pathogens (de los Reyes, 2010). Stable foams require three components, air bubbles, surfactants and hydrophobic particles (bacterial cells). With only air bubbles and surfactants, a common feature of plant startup, a non-persistent superficial white foam is generated, while in the absence of surfactants, an oily surface scum is generated (Petrovski et al., 2011). Most foams become stabilized by presence of high numbers of filamentous hydrophobic bacteria, but theoretically any sufficiently hydrophobic cells, including unicells will stabilize them (de los Reyes, 2010; Petrovski et al., 2011).

This review deals with a group of filamentous bacteria, the *Chloroflexi*, who have been associated with bulking and foaming events in activated sludge plants. Although much is known about their identity and ecophysiology in Danish EBPR plants, little information is available regarding their global distribution. This review has as one of its aims to fill that gap in our knowledge.

## The *Chloroflexi*

The phylum ‘*Chloroflexi*, embraces an ecologically and physiologically diverse group of bacteria, which have been detected in an increasingly wide range of anaerobic habitats including sediments, hot springs, methanogenic anaerobic sludge digesters where they are highly abundant and play an important fermentative role as well as contributing to sludge granulation (Hug et al., 2013; McIlroy et al., 2016; Sun et al., 2016; Xia et al., 2016; Petriglieri et al., 2018; Bovio et al., 2019), the human oral cavity (Campbell et al., 2014), Anammox reactors (Kindaichi et al., 2012; Cao et al., 2016; Wang et al., 2016) and activated sludge communities (Björnsson et al., 2002; Kragelund et al., 2007a, 2011; Speirs et al., 2009, 2011, 2017; Yamada and Sekiguchi, 2009; Hanada, 2014; McIlroy et al., 2018; Andersen et al., 2019; Nierychlo et al., 2019). This is not the place to explore in detail their still evolving systematics, which have been dealt with elsewhere (Hanada, 2014). So only a brief overview is provided here.

It soon became apparent that the original name used to describe these, the Green non-sulfur bacteria’ was inappropriate, as they included a diverse range of mesophilic and thermophilic aerobic and anaerobic chemoorganoheterotrophs as well as photolithoautotrophic bacteria (Hanada, 2014). It was also soon recognized that they were not closely related phylogenetically to the *Chlorobia* (Green Sulfur Bacteria) or the Purple Non-Sulfur Bacteria. Hence the phylum name *Chloroflexi* proposed by Garrity et al. (2001) is now accepted.

Based on the 16S rRNA gene sequences of the then available strains, a single class, the ‘*Chloroflexi*,’ containing two orders, the *Chloroflexales* and the *Herpetosiphonales* was proposed by them. All known members were filamentous with an unusual gliding mechanism as a means of motility, and although most stained Gram negatively, none had the characteristic lipopolysaccharide outer membrane of the Gram-negative bacteria. Only four *Chloroflexi* genera were recognized by Garrity et al. (2001), although it was becoming clear from 16S rRNA gene sequence data retrieved from a wide range of habitats that many uncultured strains existed.

This scheme was modified by Hugenholtz and Stackebrandt (2004) to accommodate the 16S rRNA gene sequence data from newly described strains and those initially classified incorrectly. They proposed four new classes, the *Anaerolineae*, the *Dehalococcoidetes*, the *Chloroflexia* and the *Thermomicrobia*, corresponding to their earlier groupings labeled [1], [2], [3], and [5], respectively (Hugenholtz et al., 1998). Their phylogenetically distinct group 4 contained only uncultured strains, and so was not named formally. Characterization of three new isolates led Yamada et al. (2006) to propose that the *Anaerolineae* should be subdivided into two classes, the obligately anaerobic *Anaerolineae* and the aerobic or facultatively anaerobic *Caldilineae*, as delineated earlier by Sekiguchi et al. (2003).

These classifications have persisted, and so based on 16S rRNA genesequence data, the phylum *Chloroflexi* was thought by Hanada (2014) to contain at least six classes. They are the *Chloroflexia*, which includes all the photolithotrophic members, the *Anaerolineae*, the *Caldilineae*, the *Ktedonobacteria*, all of which appear to be multicellular filamentous bacteria, and the *Dehalococcoidetdia.* The *Thermomicrobia*, all members of which were thought to be thermophilic, were once considered to belong to a separate phylum by Garrity et al. (2001). In 2013, Kawaichi et al. (2013) proposed that a new class, the *Ardenticatenia*, was needed to accommodate a strain they isolated from an iron rich coastal hydrothermal field. This addition was not included in the review of Hanada (2014), and nor was that of Dodsworth et al. (2014), based on their new strain *Thermoflexus hugenholtzii.* While its 16S rRNA genesequence showed it to be a member of the phylum *Chloroflexi*, it was not embraced by any of the then six existing classes, and so a new class, the *Thermoflexi*, was proposed to accommodate it. It seemed then probable that further modifications to the classification of these organisms would be necessary as more strains were characterized using novel characters, and some of these have already suggested the current schemes for *Chloroflexi* systematics need modifications.

Thus, phylogenetic markers other than 16S rRNA gene sequences have been used. For example, Kunisawa (2011) used genome sequence order comparisons, which reveal shared ancestry between populations. His data gave similar outcomes to those using 16S rRNA gene sequence data, and confirmed that the *Thermomicrobia* were not a separate phylum, but a class within the *Chloroflexi.* On the other hand, the application of alternative molecular markers, the signature indels in conserved proteins of the *Chloroflexi* by Gupta et al. (2013) gave a phylogeny markedly different to those based on 16S rRNA gene sequences. Protein sequences from each of their clades each possessed distinctive indels, but overall their patterns revealed that the phylum *Chloroflexi sensu stricto* should contain only members of the classes *Chloroflexia* and the *Thermomicrobia*, while the other four classes of Hanada (2014) should be considered as taxa related to, but not part of the phylum *Chloroflexi*. More recently Parks et al. (2018) have looked at possible evolutionary and phylogenetic relationship among the *Chloroflexi* with their Genome Taxonomic Database (GTDB), based on concatenated alignments of 120 single copy marker genes, and taxa constructed using relative sequence divergence. Among other changes, they suggest that the taxa classes of *Caldilineae, Ardenticatenia* and *Thermoflexia* in the SILVA/MiDAS classification should be relegated to the orders *Caldilineales, Ardenticatenales* and *Thermoflexales*, respectively. The scheme has already led, for example, to marked changes in the proposed classification of *Ca.* ‘Amarolinea aalborgensis’ (Andersen et al., 2019), as discussed below.

## The Bulking and Foaming Filamentous Bacteria

Originally characterized exclusively on their microscopic features and staining reactions, Eikelboom (1975) separated activated sludge bulking and foaming filaments into several individual ‘morphotypes’ based on their morphology and staining reactions, in the absence then of more discerning methods for their identification. He was unable to culture and appropriately characterize most of these, and the ‘naming’ system he used for them does not follow the strict rules of the International Code for Bacterial Nomenclature. Instead he adopted a morphotype based numerical system (e.g., types 0092, 0851, 0803, etc.), an imprecise system of labeling but one still used widely, inevitably leading to confusion and communication compromises among microbiologists interested in these bacteria.

Furthermore, his widely used morphology based identification manuals (Eikelboom and Van Buijsen, 1983; Eikelboom and Geurkink, 2002; Eikelboom et al., 2006) and those of Jenkins et al. (2003) do not always agree about which feature/s are diagnostic for each filament morphotype. The manuals are also incomplete, and so the more recently characterized filaments including several *Chloroflexi* (Speirs et al., 2009, 2011, 2017; Andersen et al., 2019; Nierychlo et al., 2019) and “*Candidatus* Villigracilis,” which data here show (Supplementary Data File S1) is clearly an abundant filament common to activated sludge systems around the world are missing.

Those basing their filament ‘identifications’ exclusively on characters listed in these manuals assume the listings embrace all existing activated sludge filaments. So inevitably they try to match their unknown to one of the filaments described there, even though the match may not be close, and their filament may not be mentioned. This microscopic approach, which is still popular, is quick and simple with practice but uses too few characters, and has one major flaw. It is clear now that a single Eikelboom morphotype may embrace several phylogenetically very different bacteria, which are indistinguishable under the microscope (Seviour, 2010a, b). As the *Chloroflexi*, like most other filaments, possess no unique morphological feature/s to allow their precise identification in this manner, microscopy alone has failed to reveal whether any of these filament morphotypes are members of this phylum.

During this period, some of the bulking and foaming filament morphotypes of Eikelboom (1975) were cultured successfully, often after micromanipulation, and their 16S rRNA genes sequenced (Nielsen et al., 2009b; Seviour, 2010b; Yoon et al., 2010; Nielsen and McMahon, 2014). Obtaining this information is straightforward for the relatively few filaments isolated into pure culture (see below), but the likelihood of growing more of these filaments, in the absence of any information on their individual growth requirements, seems increasingly small (Seviour, 2010b). The sequence data generated reveal that most bulking filaments represent previously undescribed bacteria. Other filaments have emerged as filamentous forms of previously identified unicellular bacteria, a situation also likely to make their identification by microscopy problematic. Examples are given in Seviour and Nielsen (2010). Consequently, any applied control methods based solely on their microscopic identification are unlikely to work reliably.

The *Chloroflexi* cultured from activated sludge include a *Herpetosiphon* sp. (Bradford et al., 1996), which was thought unlikely to cause bulking (Nielsen et al., 2009b), and has not been characterized further. Of the *Chloroflexi* filaments in activated sludge, “*Ca*. Kouleothrix aurantiaca.” has been grown and characterized phenotypically (Beer et al., 2002; Kohno et al., 2002; Kragelund et al., 2007a), as has a *Chloroflexi* filament isolated in Korea (Yoon et al., 2010), named here as a “*Ca.* Defluviithrix” (see later). No filament in the two identification manuals is a close morphological match to the isolate of Yoon et al. (2010). Earlier claims of success in growing the *Chloroflex*i morphotype type 0092 (Horan et al., 1988; Buali and Horan, 1989; Bradford et al., 1996) should be viewed cautiously. No convincing evidence has been presented that the filaments they cultured were of this morphotype, and Speirs et al. (2009) showed that the 16S rRNA targeted FISH probe designed by Bradford et al. (1996) to target their type 0092 *Bacteroidetes* isolate, did not target the common type 0092 morphotype seen *in situ* in Australia or elsewhere.

## Microscopy Based Survey Data

Early plant surveys depended by necessity on using these morphological/staining characters in attempts to see which filaments dominated plants profiles. In most of these (summarized by Seviour and Nielsen, 2010; Rossetti et al., 2017), usually only single samples have been taken, often from a single point in the treatment process, and in many cases from a small selection of treatment plants. The plants sampled are usually located considerable distances from each other, treating a wide diversity of undefined influents, and the filament ‘identifications’ were rarely complemented by comprehensive and detailed operational data (Seviour, 2010b; Rossetti et al., 2017). These compromises limit the usefulness of such survey data, providing as they do a limited snapshot of the filaments present at a single point in time.

Early microscopic surveys by Eikelboom and Van Buijsen (1983), and Jenkins (1992) of European and North American treatment plants respectively, attempted to see if any correlation existed between individual filament morphotypes and plant operational conditions. Their ‘rules of thumb’ are still used widely in attempts to solve bulking and foaming problems in large scale plants around the world, despite many of these relationships being questioned in surveys using molecular methods, as discussed later. More recent survey data (Rossetti et al., 2017) in general support many of their conclusions, but not all similar studies agree (Seviour et al., 1994).

## Impact of Molecular Tools on Understanding the Bulking and Foaming Filaments

### Which *Chloroflexi* Filaments Are Present in Activated Sludge Plants?

Most bulking plant communities contain a low biodiversity of filamentous bacteria, among which are the one or sometimes more dominant populations responsible for sporadic incidents of bulking. It is now clear that these communities commonly contain several filamentous members of the phylum *Chloroflexi* (McIlroy et al., 2015, 2018; Andersen et al., 2019; Nierychlo et al., 2019), and FISH probes, together with helper and competitor probes where employed, have been designed for many of these (Table 1). In writing this review and taking into consideration the recent literature, it was decided that the Eikelboom filaments characterized with molecular methods should no longer be recognized solely by their numerical designations. Thus, together with the phenotypic characters listed in the identification manuals of Jenkins et al. (2003), Eikelboom et al. (2006), and Seviour and Nielsen (2010), it now seems an appropriate time to provide the *Chloroflexi* filaments with provisional or *Candidatus* names where appropriate. Switching to a system of filament nomenclature based on using valid names was adopted by McIlroy et al. (2015), and has become the convention used subsequently by McIlroy et al. (2017a), Speirs et al. (2017), Andersen et al. (2019), and Nierychlo et al. (2019).

**TABLE 1:** FISH probes, oligonucleotide sequences, target coverage detail and hybridization conditions to target members of the filamentous *Chloroflexi* reported in activated sludge.

**Probe**	**Target group**	**Eikelboom Morphotype**	**Formamide (%)**	**Probe sequence (5′–3′)**	**Competitor sequence (5′–3′)**		**Helper probes (5′–3′)**		**Coverage°**	**Non-target hits°**	**References**
**Domain level probes**											
EUB338 I	Most *Bacteria*	NA	0–60	GCT GCC TCC CGT AGG AGT	NA	NA	NA	NA	ND	ND	Amann et al. (1990)
EUB338 II	Most *Planctomycetales*	NA	0–60	GCA GCC ACC CGT AGG TGT	NA	NA	NA	NA	ND	ND	Daims et al. (1999)
EUB338 III	Most *Verrucomicrobiales*	NA	0–60	GCT GCC ACC CGT AGG TGT	NA	NA	NA	NA	ND	ND	Daims et al. (1999)
**Phylum and higher clade level probes**											
GNSB941	Most members of the *Chloroflexi*	NA	35	AAA CCA CAC GCT CCG CT	NA	NA	NA	NA	ND	ND	Gich et al. (2001)
CFX1223	Most members of the *Chloroflexi*	NA	35	CCA TTG TAG CGT GTG TGT MG	NA	NA	NA	NA	ND	ND	Björnsson et al. (2002)
CFX109	Subgroup 3 of the *Chloroflexi*	NA	30	CAC GTG TTC CTC AGC CGT	NA	NA	NA	NA	ND	ND	Björnsson et al. (2002)
CFX784	Subgroup 1 of the *Chloroflexi*	NA	30	ACC GGG GTC TCT AAT CCC	NA	NA	NA	NA	ND	ND	Björnsson et al. (2002)
CFX1A331	Members of the class *Caldilineae*	NA	30	CCC CGT AGG AGT CGG GAC	NA	NA	NA	NA	ND	ND	Yoon et al. (2010)
Caldi0678	Most members of the class *Caldilineae*	NA	30	TTC CAC CAC TAC ACC GGG	Comp1-Caldi-0678	TTT CAC CAC TAC ACC GGG	NA	NA	ND	ND	Kragelund et al. (2011)
					Comp2-Caldi-0678	TTC CAC CGC TAC ACC GGG					
**Phylotype targeted probes**											
AHW183	‘Nostocoida limicola’-like filament	N. limII	35	CCG ACA CTA CCC ACT CGT	NA	NA	NA	NA	ND	ND	Schade et al. (2002)
CHL1851	“*Ca*. Kouleothrix”	1851	35	AAT TCC ACG AAC CTC TGC CA	NA	NA	NA	NA	6/20	0	Beer et al. (2002)
EU25-1238	“*Ca*. Kouleothrix”	1851	35	CTG CGC ATT GCC ACC GAC AT	NA	NA	NA	NA	4/20	0	Kragelund et al. (2007a)
T1851-2	“*Ca*. Kouleothrix”	1851	NA	CCT GAG CGT CAG ATA TGG CC	NA	NA	NA	NA	2/20	0	Guo and Zhang (2012)
CFX197	“*Ca*. Promineofilum”	0092	40	TCC CGG AGC GCC TGA ACT	CFX197 comp	TCC CGA AGC GCC TGA ACT	NA	NA	26/34	0	Speirs et al. (2009)
CFX223	“*Ca*. Promineofilum”	0092-like	35	GGT GCT GGC TCC TCC CAG	NA	NA	CFX223 H202	AGC GCC TGA GCT TTC AGT CAT C	2/34	0	Speirs et al. (2009)
CFX67mix^#^	“*Ca*. Sarcinithrix”	0914	35	TTC CGA AGA TYA GGT TCG	CFX67 comp	TTC CGA AGA TCG GGT TCG	CFX67-H46	TTC GAC TTG CAT GTG TTA RGC	6/13	0	Speirs et al. (2011)
							CFX67-H95	CCG TRC GCC ACT AAC CYT			
CFX449	“*Ca*. Sarcinithrix”	0914	50	GGG ATA CCG TCC TTG TCT CT	CFX449C1_comp	GGG GTA CCG TCC TTG TCT CT	CFX449_H1	ACG TAG TTA GCC GAG ACT TAT TCC T	12/13	1	Nierychlo et al. (2019)
							CFX449_H2	TCT CCC AGA AAA GRR GTT TAC GAC CCG			
CFX1151	“*Ca*. Sarcinithrix”	0914	50	TTG ACT CCG GCA GTC CCA CT	CFX1151_C1	TTG ACA CCG GCA GTC CCA CT	CFX1151_H1	ATC CCC ACC TTC CTC CGG T	12/13	1	Nierychlo et al. (2019)
							CFX1151_H2A	TAA CTA GTA GBG AGG GTT GCG CTC GT			
							CFX1151_H2B	TAA CTA GTA GCA GGG GTT GCG CTC GT			
CFX64	“*Ca*. Amarolinea”	0092-like	30	TCT ACC TAA GCA GAC CGT TC	NA	NA	CFX64_H1	AAC TTG CAT GTG TTA AGC ACG CC	1/2	0	Nierychlo et al. (2019)
							CFX64_H2	TCA CCC GTG CGC CAC TG			
CFX763A	“*Ca*. Villigracilis” – sub-group A	Unk.	45	GTT TAC TAC CCT AGC TTT CGC	CFX763A_C1	GTT CAC TAC CCT AGC TTT CGC	CFX763AB_H1A	TAG GAT TAC CGG GGT CTC TAA TCC C	68/260	1	Nierychlo et al. (2019)
					CFX763A_C2	GTT TAC TCC CCT AGC TTT CGC	CFX763AB-H1B	TAG GAT TAC CSG GGG TCT CTA ATC CC			
					CFX763A_C3	GTT TGC TAC CCT AGC TTT CGC					
					CFX763_C4	GTT TAC TAC CCT AGC TGT CGC					
CFX763B	“*Ca*. Villigracilis” – sub-group B	Unk.	45	GTT TAC TAC CCT AGC TGT CGC	CFX763B_C1	GTT TAC TAC CCT AGC TTT CGC	Same as CFX763A	Same as CFX763A	91/260	0	Nierychlo et al. (2019)
CFX998	“*Ca*. Trichobacter”	0803	50	CAG ATC ACT ACC ACC GTC	CFX998comp	CAG ATC ACT ACC ACC AGA ACC	NA	NA	2/2	0	Speirs et al. (2015)
CFX841	Filamentous *Ardenticatenia* sp.	ND	30	AGC ACA GAA GGT CTT ACG	NA	NA	CFX841 H1	ACC TCC TAC GCC TAG TTG	ND	ND	Speirs et al. (2015)
T0803ind-0642	“*Ca*. Defluviifilum”	0803	30	CTG CCT CAA GCT ACT CAG	NA	NA	h1 T0803ind-0607	AGT TAA GCC AGG AGA TTT	3/3	0	Kragelund et al. (2011)
							h2 T0803ind-0625	TTT CCA ACG ACC CCT CCC			
							h3 T0803ind-0662	GAA TTC TAC ACC CCT CTC			
							h4 T0803ind-0680	ATT CCA CCA CTA CAC CGG			
CFX86mix	“*Ca*. Catenibacter”	0041	35	CCG CCA CTT TCA RGG ATA C	NA	NA	CFX86_H1	AWG TAC CCY CTC ACG TTC GAC	7/9	3	Speirs et al. (2017)
							CFX86_H2	WCC TAC GTS TTA CKC ACC CGT			
CFX194mix	“*Ca*. Amarithrix”	0675	45	GCG CCA GAG CTT TCC CCA + GCA CCA GRG CTT TCC CCA	CFX194-comp1	GCG CCA GAG CTT TCC CCC	CFX196_H1	CAT CTC TTC CCA GAA ATA TGG ATC TAT G	4/7	0	Speirs et al. (2017)
					CFX194-comp2	CCG GCA GAG CTT TCC CCA	CFX196_H2	CGG AYG CAG ACC CCT CCY RRA			
Ntlc439	*Nitrolancea hollandica*	NA	40	TTG CTT CGT CCC CCA CAA	cNtlc439	TTG CTAT CGT TTA CTG CTC	NA	NA	ND	ND	Sorokin et al. (2012)
Ntlc-804	*Nitrolancea hollandica*	NA	40	CAG CGT TTA CTG CTC GGA	c1Ntlc c2Ntlc c3Ntlc804 c4Ntlc804	CAG CGT TTA CTG CGC GGA CAT CGT TTA CTG CTC GGA CAG CGT TTA CTG CTA GGA CAG CGT TTA CTG CTA GGA	NA	NA	ND	ND	Sorokin et al. (2012)

*See original publications for further hybridization details. ND = not determined. ^#^This probe has been superseded by CFX449 and CFX1151 (Nierychlo et al., 2019). °Coverage values determined using the SILVA123 (Quast et al., 2013) and modified MiDAS 2.1 taxonomy (McIlroy et al., 2017b) databases, and ARB (Ludwig et al., 2004). NA = not applicable.*

In addition to the cultured “*Ca*. Kouleothrix spp.” mentioned above (Beer et al., 2002; Kohno et al., 2002; Kragelund et al., 2007a), are the uncultured “*Ca*. Promineofilum breve” (type 0092) (Speirs et al., 2009; McIlroy et al., 2016), “*Ca.* Defluviifilum” (Danish type 0803) (Kragelund et al., 2011), “*Ca.* Trichobacter” (Australian type 0803) (Speirs et al., 2015), “*Ca.* Catenibacter” (type 0041) (Speirs et al., 2017), “*Ca*. Amarithrix” (type 0675) (Speirs et al., 2017), “*Ca*. Villigracilis” (not associated with any Eikelboom morphotype) (Nierychlo et al., 2019), and “*Ca*. Amarolinea aalborgensis,” an atypical type 0092 (Andersen et al., 2019; Nierychlo et al., 2019; Table 1). Attributes of these individual filaments are discussed in detail later in the review.

### What Have FISH/PCR Cloning Data Revealed About *Chloroflexi* in Activated Sludge and Their Impact on Plant Operations?

One of the earliest reports of *Chloroflexi* in activated sludge was that of Juretschko et al. (2002) in an industrial plant removing N. After PCR/cloning, they recovered 15 *Chloroflexi* clones, whose sequences placed them all in the Group 1 of Hugenholtz et al. (1998). In the absence then of suitable FISH probes, they could not be identified further. Again in 2002, Björnsson et al. (2002) using FISH analyses, suggested the filamentous *Chloroflexi* were ubiquitous in non-bulking activated sludge and especially abundant in plants designed to remove N and in some cases P. On the basis of their 16S rRNA gene sequence data, most were members of subgroup 3 (now containing members of the class *Chloroflexia*). They designed and validated several FISH probes based on their 16S rRNA gene sequences, and some of these are still widely used today. They are the phylum targeted probe CFX1223, the subgroup 3 probe CFX109 and subgroups 1a and 1b targeted probe CFX784 (Table 1). It was recommended that the CFX1223 and the GNSB941 *Chloroflexi* phylum probes designed earlier by Gich et al. (2001), should be used with them, a practice still followed routinely. Björnsson et al. (2002) also reported for the first time that some of the *Chloroflexi* in full-scale plants failed to respond to the EUBmix probes designed to target all *Bacteria.* Unfortunately they were unable to relate their FISH positive *Chloroflexi* filaments to any of the morphotypes of Eikelboom (1975), because of their location within the flocs. However, a reassessment here of these clone sequences using the MiDAS 2.1 database can now reveal that the Björnsson et al. (2002) and Juretschko et al. (2002) *Chloroflexi* clone sequences AF234698, AF234710, AF234759, and X84472 were all derived from “*Ca*. Villigracilis,” while the Björnsson et al. (2002) clone sequence X84565 is here identified as from a “*Ca*. Kouleothrix.”

Most published FISH surveys have been relatively small scale, and usually carried out on single plant communities, or commonly performed to validate newly designed FISH probes targeting a known individual filament population/s e.g. (Beer et al., 2002; Björnsson et al., 2002; Kragelund et al., 2007b, 2011; Speirs et al., 2009, 2011, 2015, 2017; Nittami et al., 2017; Andersen et al., 2019; Nierychlo et al., 2019). These have often revealed possible relationships between filament abundances and plant configurations, but not always detailed individual plant operational conditions. The general conclusions from these studies are that the *Chloroflexi* in activated sludge plants are ubiquitous and almost exclusively filamentous. In non-bulking biomass samples they are usually located substantially within the floc, where, as stated earlier, they provide the matrix around which the floc material aggregates (Nielsen et al., 2009b). FISH probing has revealed that some including *Ca* ‘Amarolinea,” “*Ca*. Kouleothrix,” “*Ca.* Amarithrix,” “*Ca*. Defluviithrix,” “*Ca.* Sarcinithrix,” and “*Ca.* Catenibacter” can extend into the mixed liquor and form interfloc bridges (Beer et al., 2002; Speirs et al., 2011, 2015, 2017; Nierychlo et al., 2019).

Yet Wagner et al. (2015) have questioned the role of the *Chloroflexi* in bulking. Although showing in their qFISH based study that the *Chloroflexi* were located predominately protruding from the floc surfaces, modeling showed they were less important than the floc bound actinobacterial “*Ca.* Microthrix parvicella” in determining floc settlabilities. However, only phylum level CFX mix probes were used for *Chloroflexi* detection and quantification, so no attempt was made to identify which individual *Chloroflexi* populations might be present in their community. This concern is especially applicable to those mentioned above, whose bundles of filaments act to join flocs together (e.g., Beer et al., 2002), as to whether they were present in the communities studied. Thus, the assumption that all the filamentous *Chloroflexi* behave in activated sludge as Wagner et al. (2015) describes clearly requires further examination.

According to flotation theory, any hydrophobic cell can stabilize the foams appearing on the surface of activated sludge aerobic reactors (Petrovski et al., 2011), and explains why those of the actinobacterial Mycolata and “*Ca*. Microthrix parvicella” (de los Reyes, 2010) are commonly seen there. This theory may explain the frequently reported presence of “*Ca.* Promineofilum” filaments (Rossetti et al., 2017) in foams. However, de los Reyes (2010) has suggested this filament is an *‘accidental’* foam former, carried there by hydrophobic biomass. Furthermore, McIlroy et al. (2016) showed with their genomic data that “*Ca.* P. breve” cells are not hydrophobic, and nor are those of “*Ca*. A. aalborgensis” (Andersen et al., 2019) and the filaments described by Nierychlo et al. (2019), a decision based on *in situ* MAC (microsphere adsorption to cells) assays. However, those of “*Ca.* Defluviifilum” are, which may explain why these filaments have been observed often in foams, where they probably assist in their stabilization (Kragelund et al., 2011).

As mentioned earlier, many of these *Chloroflexi* filaments appear to prefer EBPR plants (see earlier), which generally operate at long sludge ages. This characteristic might suggest these are slow growing bacteria, being washed out at shorter sludge ages. Alternatively, because most are associated intimately with flocs, they will be recycled with the settled RAS, and thus stay within the EBPR plants, only leaving in the wasted sludge. Their high abundances may suggest their ecophysiology provides them with some competitive advantage over other bacterial populations in response to the strong selective pressures exerted on the EBPR bacterial community (see earlier). These may include an ability to assimilate substrates anaerobically and use them for synthesis of storage compounds which are then available aerobically for energy production and growth, as seen with the GAO and PAO phenotype (Seviour et al., 2003; Oehmen et al., 2007), or to perform fermentation and/or anaerobic respiration as reported by McIlroy et al. (2016), Andersen et al. (2019), and Nierychlo et al. (2019). Such a facultatively anaerobic chemoheteroorganotrophic lifestyle would provide them with a selective advantage in EBPR plants, where biomass is cycled continuously between anaerobic and aerobic zones (see earlier), and possibly render them less competitive in fully aerobic systems, where they are less common (McIlroy et al., 2016).

Previously undescribed filamentous *Chloroflexi* members of the *Anaerolineae* have been detected at high abundances in anaerobic digesters, systems used to stabilize wasted biomass from plants, and to generate methane as an energy source (St-Pierre and Wright, 2014; Kirkgaard et al., 2017; McIlroy et al., 2017a; Petriglieri et al., 2018). It seems reasonable to assume that at least some of these *Chloroflexi* derive from the aerobic activated sludge community, and consequently might support biomass bulking and foaming there. Kirkgaard et al. (2017) proposed that the *Chloroflexi* in their digesters consisted mainly of populations migrating from the aerobic bioreactors, although many they identified there were exclusive to the digesters. Petriglieri et al. (2018) also showed that members of the *Chloroflexi* were highly abundant in digesters, with most of the populations identified there being autochthonous, and thus probably having important roles in sludge digestion.

The question is whether these migrating *Chloroflexi* can survive and grow in these digesters, being facultative anaerobes (see above), and what their role might be in stabilizing foam formation commonly seen there, and, in having a filamentous morphology, in sludge granule formation. Foam stabilization seems unlikely because most of these flaments are not hydrophobic (Nierychlo et al., 2019). By comparing amplicon 16S rRNA gene sequencing abundances where both cellular and exocellular DNA fragments would be quantified, with those estimated by qFISH, a method requiring intact metabolically active cells, they suggested that the migrating *Chloroflexi* population steadily decreased in abundance in the digesters. In some cases they were barely detectable by either of these analytical methods. However, their naked DNA appears to survive there, to be detected by amplicon sequencing. These trends would suggest that the migrating *Chloroflexi* are not metabolically highly active populations in these digesters, and probably play a minor role in sludge stabilization and breakdown. Similar data from other countries are needed to confirm this hypothesis.

### How Abundant Are the *Chloroflexi* in Activated Sludge Plants From FISH Analyses

The most popular FISH quantitative protocol is to express the data as population relative abundances where the biovolume of cells responding to the FISH probe for the filament population of interest is expressed as a percentage of cells responding to the EUBmix FISH probes designed to cover all members of the domain *Bacteria* (Daims et al., 1999). Unfortunately it is clear that some of the *Chloroflexi* filaments in activated sludge, and c.a 20% of all *Chloroflex*i 16S rRNA gene sequences in the SILVA128 NR99 database do not possess a perfect match to any of the domain targeted EUBmix probes (Morgan-Sagastume et al., 2008; Nielsen et al., 2009b; Speirs et al., 2009; Kragelund et al., 2011). However, not all probe target mismatches will be sufficiently destabilizing to prevent probe hybridization (Table 2), as appears to be the case with “*Ca*. Defluviifilum” (Nierychlo et al., 2019). An overview of the individual EUBmix probe target sites and the corresponding probe sequences in the *Chloroflexi* filaments described here are given in Table 2. Resolving these problems by designing more EUBmix FISH probes to cover all the known *Chloroflexi* 16S rRNA target site variants is not straightforward.

**TABLE 2:** Theoretical hybridization efficiency for the EUB338 I, II, III FISH probes, and target sites possessing mismatches within those *Chloroflexi* filament phylotypes defined by their respective FISH probes (see Table 1).

**Phylotype**	**Phylotype specific FISH probe**	**MiDAS 2.1 (amended) sequences targeted by phylotype FISH probe/total MiDAS 2.1 (amended) phylotype sequences**	**EUB338 version with the least destabilizing mismatches**	**Hybridization efficiency (kcal/mol)**				**Hybridization efficiency (%)^∗^**	**Melting [Formamide] (%)**	**Putative FISH response^ø^**
							
				**ΔG°_1_**	**ΔG°_2_**	**ΔG°_3_**	**ΔG°_*overall*_**			
“*Ca*. Amarithrix”	CFX194mix	4/7	EUB338-I	−23.6 (0.0)	−1.6 (0.0)	−5.2 (0.5)	−16.7 (0.5)	1.00 (0.00)	+50.9 (1.1)	+
“*Ca*. Defluviiflium”	T0803ind-0642	3/3	EUB338-I	−23.6 (0.0)	−1.6 (0.0)	−6.1 (1.1)	−15.8 (1.1)	0.99 (0.00)	+48.6 (2.7)	+
“Ca. Defluviithrix”	NA	0/7	EUB338-I	−23.6 (0.0)	−1.6 (0.0)	−5.5 (0.6)	−16.4 (0.6)	0.99 (0.00)	+50.3 (1.4)	+
“*Ca*. Catenibacter”	CFX86mix	10/12	EUB338-I	−23.6 (0.0)	−1.6 (0.0)	−5.8 (0.3)	−16.1 (0.3)	1.00 (0.00)	+49.6 (0.7)	+
“*Ca*. Promineofilum”	CFX197	29/34	EUB338-III	−17.6 (0.5)	−1.9 (0.0)	−4.6 (0.8)	−11.0 (1.0)	0.88 (0.20)	+15.9 (8.8)	–
“*Ca*. Trichobacter”	CFX998	2/2	EUB338-III	−18.1 (0.0)	−1.9 (0.0)	−5.1 (0.0)	−11.1 (0.0)	0.95 (0.00)	+18.5 (0.0)	–

*Values are mean determinations against multiple target sequences, with the standard deviation in parentheses. Calculations determined using the mathFISH (Yilmaz and Noguera, 2004; Yilmaz et al., 2011, 2006) tool with the following parameters: temperature 46°C, [NaCl] 0.9M, [probe] 500nM. EUB338 variety chosen for each phylotype was that possessing the least destabilizing mismatch, determined using the mathFISH Formamide curve generator (Yilmaz and Noguera, 2007). For FISH probe details see Table 1. ^∗^Hybridization efficiency at 0% [Formamide]. ^*Ø*^Putative FISH response based on the calculated hybridization efficiency at 30% formamide. Most members of the “*Ca*. Villigracilis” (see Supplementary Table S1), “*Ca*. Sarcinithrix,” “*Ca*. Kouleothrix,” “*Ca*. Amarolinea,” and N. limI I-like *Chloroflexi* filaments (Table 1) possess perfect matches to one of the EUB338 FISH probe variants (Amann et al., 1990; Daims et al., 1999).*

Most FISH filament analyses show how widely distributed the *Chloroflexi* are in wastewater treatment plants, although globally the numbers of plants examined are still small. Thus, in a bulking plant in Poland receiving abattoir and dairy milk wastes and designed to remove N and P, Miłobędzka and Muszyński (2015) showed that the *Chloroflexi* dominated the mixed liquor filament populations (57% of the total bacterial community). Among these, “*Ca.* Defluviifilum” (Kragelund et al., 2011) were more abundant than “*Ca*. Kouleothrix” and “*Ca*. Promineofilum,” but not too surprisingly then, about half of all their *Chloroflexi* could not be identified below phylum level. In later studies (Miłobędzka et al., 2016) they tried to relate operational parameters like sludge age to abundances of those filaments for which FISH probes were available. Not all these findings agreed with data generated in larger surveys in other countries, probably because their studies were based on sporadic sampling and short survey times, conditions under which such relationships are not always revealed convincingly (Mielczarek et al., 2012).

Filamentous bacteria emerging from aerobic granular surfaces have been identified by FISH over an extended period of operation of reactors supplied with different feeds (Figueroa et al., 2015). These too showed a predominance of *Chloroflexi* filaments in reactors, especially those fed synthetic wastewater and waste from a fish cannery processing plant, but not in those fed processed marine products. However, as only the phylum level *Chloroflexi* CFX1223/GNSB941 probes were used, no further taxonomic information was generated. Earlier Morgan-Sagastume et al. (2008) had used only phylum level *Chloroflexi* probes to detect their presence in a nutrient removal activated sludge plant.

Clearly the most comprehensive and extended filament FISH and q-PCR based survey data published so far are those of Mielczarek et al. (2012), which followed *Chloroflexi* populations in 56 nutrient (N and P) removal plants in Denmark over 4 years. They set out to reveal if the relative abundances of individual filaments were determined by plant design and operating conditions, and their data show that members of the phylum *Chloroflexi* were among the most abundant populations, particularly “*Ca*. Promineofilum” and “*Ca.* Defluviifilum” (McIlroy et al., 2015), and especially during the summer and autumn. They were then replaced by the actinobacterial “*Ca*. M. parvicella” during winter and spring, in response to the lower mixed liquor temperatures (Rossetti et al., 2005). “*Ca*. Kouleothrix” filaments were found in most plants, but always at low relative abundances (Nierychlo and Nielsen, 2014).

Mielczarek et al. (2012) also proposed that each plant had its own unique filament community or fingerprint, which remained generally stable over the sampling period. No evidence suggested that any relationship existed between filament relative abundances and plant operating parameters or wastewater composition over the long term, raising questions about the conclusions reached in the reports discussed above, which were based on small numbers of plants surveyed, limited samples and short operational durations. As reported elsewhere, many of their filaments hybridizing with the CFX1223/GNSB941 probes could not be identified further using the then available targeted *Chloroflexi* probes, again highlighting the probability that activated sludge communities contain a higher biodiversity, as Speirs et al. (2015, 2017), Andersen et al. (2019), and Nierychlo et al. (2019) have since shown.

A later examination of 128 samples taken from 16 Portuguese plants over a 2 year period (Dos Santos et al., 2015) used FISH only to distinguish between filaments with indistinguishable microscopic morphologies, and so the data they presented were based largely on the Eikelboom/Jenkins methods, with all their limitations (see earlier). Again “*Ca*. Kouleothrix,” “Ca. Promineofilum,” and now morphotypes 0041/0675 dominated most of their bulking samples. Not unexpectedly, their FISH data exposed phylogenetic variation within a single morphotype. Their attempts to determine which, if any processing parameters were influencing individual filament abundances generated some unexpected outcomes, not the least of which was their conclusion that type 0092 alone among the filament community there was associated with bulking in their plants. This filament rarely extends from the floc surface into the bulk liquid very far, thus ensuring any interfloc bridging is a rare event, although in high abundance, it may affect negatively floc density (Speirs et al., 2009).

## Impact of Next Generation Sequencing on Understanding the Biodiversity of the *Chloroflexi* in Activated Sludge

With the introduction of next generation DNA sequencing (NGS), it became feasible to sequence simultaneously many individually tagged DNA samples quickly and relatively inexpensively (Karst et al., 2016; Hugerth and Andersson, 2017; Nierychlo et al., 2019). It provides a detailed fingerprint of the community population composition and has been used to quantify individual bulking and foaming bacterial populations, as discussed below.

However, no amplicon sequencing protocol provides strictly quantitative estimates of population relative abundances where one population may possess several *rrn* operons, whose individual 16S rRNAs differ in their sequence (Sun et al., 2016; Espejo and Plaza, 2018). Often this information is not available for the filament under consideration. The limited currently available data suggest that the genomes of “*Ca*. P. breve” (McIlroy et al., 2016), “*Ca*. A. aalborgensis.” (Andersen et al., 2019), and “Ca. Kouleothrix” (Ward et al., 2018) *Chloroflexi* each possess a single *rrn* operon. Whether this holds true for other activated sludge *Chloroflexi* filaments is not known.

Furthermore, as with FISH probe design, only those populations whose 16S rRNA gene sequences have been generated and attributed to a specified population *in situ*, and subsequently included in reference databases will be identifiable. Furthermore, several taxonomic classifiers exist, based on different phylogenetic divisions and groupings, and so sequences will be classified to different phylogenetic groups depending on which classifier is used, as shown in Table 3 for the activated sludge *Chloroflexi.*

**TABLE 3:** Differences in *Chloroflexi* phylotype classification in reference taxonomies, Greengenes (DeSantis et al., 2006), SILVA (Quast et al., 2013), RDP (Wang et al., 2007; Cole et al., 2014), and MiDAS 2.1 (McIlroy et al., 2017b).

**Filament**	**Reference sequence**	**Greengenes**	**SILVA (release 132)**	**RDP (release 11)**	**MiDAS 2.1**
		Phylogenetic levels: domain, phylum, class, order, family, genus			
“*Ca*. Villigracilis”	HQ640558	*Bacteria*, *Chloroflexi*, *Anaerolineae*, envOPS12	*Bacteria*, *Chloroflexi*, *Anaerolineae*, *Anaerolineales*, *Anaerolineaceae*, uncultured	*Bacteria*, *Chloroflexi*, *Anaerolineae*, *Anaerolineales*, *Anaerolineaceae*, unclassified *Anaerolineaceae*	*Bacteria*, *Chloroflexi*, *Anaerolineae*, *Anaerolineales*, *Anaerolineaceae*, “*Candidatus* Villigracilis”
“*Ca*. Amarithrix”	JN391831	*Bacteria*, *Chloroflexi*, *Anaerolineae*, *Caldilineales*, *Caldilineaceae*, *Caldilinea*	*Bacteria*, *Chloroflexi, Anaerolineae*, *Caldilineales*, *Caldilineacea*, uncultured	*Bacteria*, *Chloroflexi*, *Caldilineae*, *Caldilineales*, *Caldilineaceae*, *Caldilinea*	*Bacteria*, *Chloroflexi*, *Caldilineae*, *Caldilineales*, *Caldilineaceae*, “*Candidatus* Defluviifilum” (amended: “*Candidatus* Amarithrix”)
“*Ca*. Defluviifilum”	HQ262530	*Bacteria*, *Chloroflexi*, *Anaerolineae*, *Caldilineales*, *Caldilineaceae*, *Caldilinea*	*Bacteria*, *Chloroflexi*, *Anaerolineae*, *Caldilineales*, *Caldilineaceae*, uncultured	*Bacteria*, *Chloroflexi*, *Caldilineae*, *Caldilineales*, *Caldilineaceae*, *Caldilinea*	*Bacteria*, *Chloroflexi*, *Caldilineae*, *Caldilineales*, *Caldilineaceae*, “*Candidatus* Defluviifilum”
“*Ca*. Defluviithrix”	EU875524	*Bacteria*, *Chloroflexi*, *Anaerolineae*, *Caldilineales*, *Caldilineaceae*, *Caldilinea*	*Bacteria*, *Chloroflexi*, *Anaerolineae*, *Caldilineales*, *Caldilineaceae*, uncultured	*Bacteria*, *Chloroflexi*, *Caldilineae*, *Caldilineales*, *Caldilineaceae*, *Caldilinea*	*Bacteria*, *Chloroflexi*, *Caldilineae*, *Caldilineales*, *Caldilineaceae*, “*Candidatus* Defluviifilum”
“*Ca*. Catenibacter”	HQ343217	*Bacteria*, *Chloroflexi*, *Anaerolineae*, *Caldilineales*, *Caldilineaceae*	*Bacteria*, *Chloroflexi*, *Anaerolineae*, *Caldilineales*, *Caldilineaceae*, uncultured	*Bacteria*, *Chloroflexi*, *Caldilineae*, *Caldilineales*, *Caldilineaceae*, *Litorilinea*	*Bacteria*, *Chloroflexi*, *Caldilineae*, *Caldilineales*, *Caldilineaceae*, uncultured *Caldilineae* (amended: “*Candidatus* Catenibacter”)
“*Ca*. Kouleothrix”	AB079641	Unclassified	*Bacteria*, *Chloroflexi*, *Chloroflexia*, *Chloroflexales*, *Roseiflexaceae*, *Roseiflexus*	*Bacteria*, *Chloroflexi*, *Chloroflexia*, *Chloroflexales*, *Chloroflexineae*, *Chloroflexaceae*, *Roseiflexus*	*Bacteria*, *Chloroflexi*, *Chloroflexia*, *Chloroflexales*, *Roseiflexaceae*, *Kouleothrix*
“*Ca*. Sarcinithrix”	GU808362	*Bacteria*, *Chloroflexi*, *Anaerolineae*, SHA-20	*Bacteria*, *Chloroflexi*, 1-20	*Bacteria*, *Chloroflexi*, *Caldilineae*, *Caldilineales*, *Caldilineaceae*, unclassified *Caldilineaceae*	*Bacteria*, *Chloroflexi*, SJA-15, 1-20, 1-20, “*Candidatus* Sarcinithrix”
“*Ca*. Amarolinea”	KC551586	Unclassified	*Bacteria*, *Chloroflexi*, *Anaerolineae*, C10-SB1A	*Bacteria*, *Chloroflexi*, *Caldilineae*, *Caldilineales*, *Caldilineaceae*, unclassified *Caldilineaceae*	*Bacteria*, *Chloroflexi*, SJA-15, C10-SB1A, C10-SB1A, “*Candidatus* Amarolinea”
“*Ca*. Promineofilum”	AB445106	*Bacteria*, *Chloroflexi*, *Anaerolineae*, DRC31	*Bacteria*, *Chloroflexi*, *Anaerolineae*, *Ardenticatenales*, uncultured	*Bacteria*, *Chloroflexi*, *Anaerolineae*, *Anaerolineales*, *Anaerolineaceae*, unclassified *Anaerolineaceae*	*Bacteria*, *Chloroflexi*, *Ardenticatenia*, 419, 2-1, “*Candidatus* Promineofilum”
“*Ca*. Trichobacter”	KP835206	Unclassified	*Bacteria*, *Chloroflexi*, *Ardenticatenia*, uncultured	Unclassified	Bacteria, *Chloroflexi*, *Ardenticatenia*, 419, 2-1, CWWC007 (amended: “*Candidatus* Trichobacter”)

Equally important in amplicon sequencing is to consider critically PCR primer choice. Of the data analyzed here, almost half (5/12) used sequences from the V3–V4 regions of the 16S rRNA gene. Single studies have targeted the V1–V2, the V3, the V4 and V4–V5 regions, while three targeted the V1–V3 region. Optimizing primer choice in such studies is rarely addressed, despite concerns that they are not all equally efficient (Hugerth and Andersson, 2017). Albertsen et al. (2015) showed convincingly that primers targeting the V1–V3 regions better reflected the abundances of *Chloroflexi* in a community by more than two- fold, compared to some of the primers targeting the V3–4 and V4 regions. Consequently any amplicon based data, including those in Supplementary Data File S1 obtained with suboptimal primers, and not targeting the V1–V3 regions, may under-represent the *Chloroflexi* present in those samples.

The methodological paper of Karst et al. (2018) describes a protocol for high-throughput 16S rRNA gene sequencing using both DNA and RNA templates. When rRNA is used no PCR amplification step is involved, and so the biases known to be associated with this are removed. This method has the capacity to generate full- length 16S rRNA gene sequences and populations missed by PCR amplification will be detected by it. However, biases in relative abundance value estimates will be introduced by differences in ribosome numbers in members of individual populations. Equally, any variations in sequences of individual 16S rRNA in a single population will affect the community biodiversity data.

### NGS Use in Activated Sludge Community Surveys

Amplicon sequencing holds the promise of helping identify many of the as yet unidentified *Chloroflexi* populations, as demonstrated convincingly for example by Nierychlo et al. (2019). Amplicon sequencing protocols regularly recover data from populations contributing as little as 0.1–0.01% of the total community, compared to the 0.5–2% limitation generally imposed by the PCR cloning approach (a value based on the generation and sequencing output from 50 – 200 cloned 16S rRNA gene sequences). Despite this, most of the currently available amplicon sequence data suggest population distributions observed generally mirror closely those revealed by the earlier molecular methods (Nielsen and McMahon, 2014; Hugerth and Andersson, 2017), and often do not reveal the true depth of community biodiversity (Karst et al., 2018).

To aid in identifying sequences of activated sludge bacteria, the MiDAS database was created (McIlroy et al., 2015, 2017b), whose aim is to consolidate all activated sludge bacteria into a single curated resource^1^. This resource provides a database for their classification, and a browser containing their curated functional attributes (McIlroy et al., 2015). Its value in identifying the activated sludge *Chloroflexi* and their ecophysiology will be demonstrated and discussed later.

As with the FISH surveys, many of the early amplicon studies were descriptive inventory fingerprinting or data generating exercises, involving single samples from small numbers of plants (Nielsen and McMahon, 2014). Nevertheless, these revealed predictably high community biodiversity richness, identifying members of the major recovered bacterial phyla, and sometimes extending considerably our understanding of certain functionally important groups, including the nitrifying bacteria, PAO and GAO (Nielsen and McMahon, 2014; Rodríguez et al., 2015; Daims et al., 2016; Ferrera and Sanchez, 2016). They also confirmed the high frequency of occurrences and often abundances of the *Chloroflexi* in plants of all configurations, but especially in EBPR nutrient removal systems. Therefore, the view was these bacteria should be recognized as core EBPR activated sludge populations, although in an extensive survey of Danish EBPR plants (Saunders et al., 2016), the *Chloroflexi* were often detected at relatively low abundances, Later examinations of the same plants (Nierychlo et al., 2019) suggested otherwise, which probably reflects the different PCR primers (see above and Supplementary Data File S1) used in the two studies (Albertsen et al., 2015).

As few of the plants examined in these studies were experiencing filamentous bulking or foaming incidents, no special attention was given to the putative bacteria responsible. Several have used amplicon sequencing to examine such communities. Almost all have been carried out in China, and so similar data from plants in other countries are lacking. One exception is the report of Dunkel et al. (2018), who used this approach to look at the 21 filamentous bacteria, including several *Chloroflexi* morphotypes, in two bulking and foaming German industrial treatment plants over a 3 month period. However, they did not take advantage of the MiDAS database for their filament identifications, instead using the less reliable filament database of Guo and Zhang (2012). They and others e.g. (Yang et al., 2017) have applied their data to suggest the operating factors thought to support the increased abundances of many of these filament clades (Dunkel et al., 2018), but generally these add little to what is known already.

**FIGURE 1:**
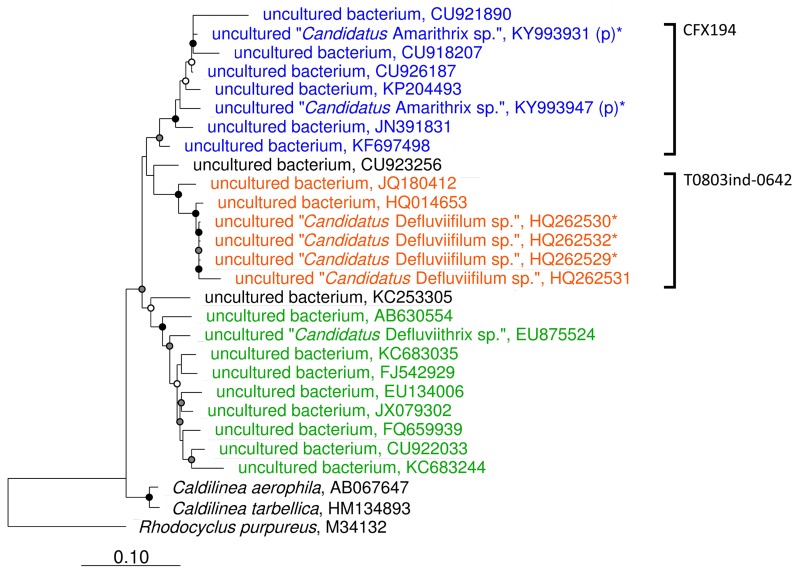
Maximum-likelihood phylogenetic tree showing the *Caldilineae* clades representing “*Ca*. Defluviifilum” (orange), “*Ca*. Amarithrix” (blue), and “*Ca*. Defluviithrix” (green). The tree was generated by ARB using the PhyMl model, and those sequences present in the MiDAS 2.1 database (McIlroy et al., 2017b). All sequences were >1200 bp in length, except for those partial sequences added using the ‘Quick add’ function in ARB, and identified by ‘(p).’ Sequences marked with ‘^∗^’ were also included as they derive from their respective filament population (Kragelund et al., 2011; Speirs et al., 2017). The scale bar corresponds to 0.1 substitutions per nucleotide. Parsimony bootstrap values were calculated as percentages of 1000 analyses, and values 50%–75% are indicated with a white circle, 76%–95% with a gray circle, and >96% with a solid black circle. Brackets to the right indicate probe coverage.

**FIGURE 2:**
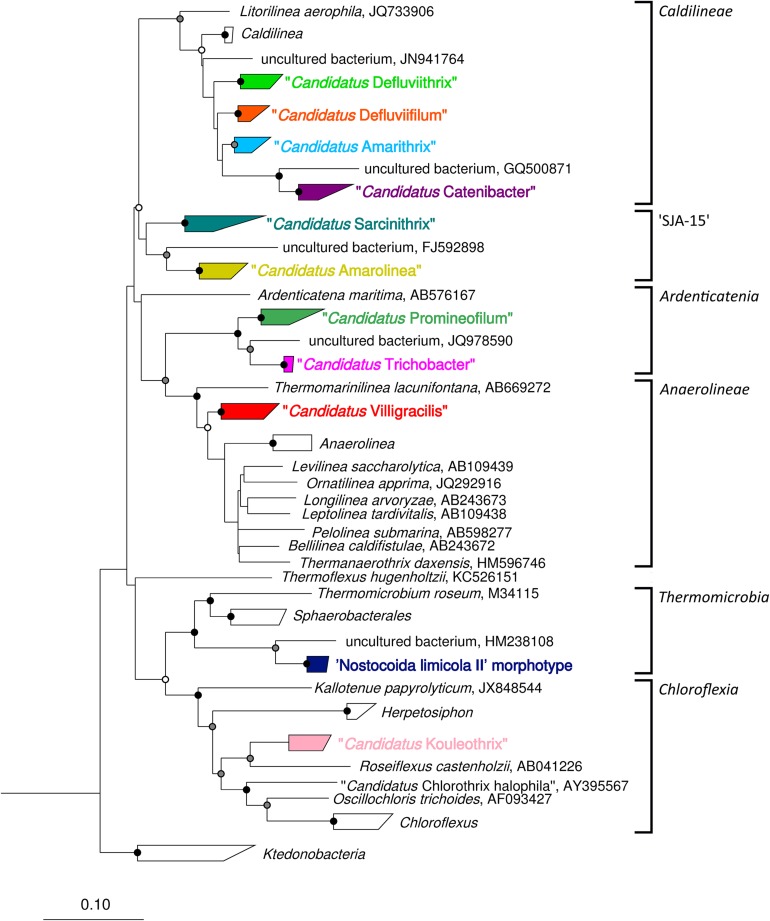
Complete maximum-likelihood phylogenetic tree for the filamentous *Chloroflexi* phylotypes reported in activated sludge. Sequences representing phylotypes are color coded: “*Ca*. Catenibacter” (purple), “*Ca*. Amarithrix” (blue), “Ca. Defluviifilum” (orange), “*Ca*. Defluviithrix” (light green), “*Ca*. Sarcinithrix (teal), “*Ca*. Amarolinea” (yellow), “*Ca*. Villigracilis” (red), “*Ca*. Trichobacter” (pink), “*Ca*. Promineofilum” (dark green), ‘Nostocoida limicola II’ morphotype (dark blue), and “*Ca*. Kouleothrix” (lilac). For more detail of the “*Ca*. Amarithrix,” “*Ca*. Defluviifilum,” and “*Ca*. Defluviithrix” clades, see Figure 1. The tree was generated by ARB using the PhyMl model, and sequences present in the MiDAS 2.1 database (McIlroy et al., 2017b). All sequences were >1200 bp in length, except for those partial sequences added using the ‘Quick add’ function in ARB. The scale bar corresponds to 0.1 substitutions per nucleotide. Parsimony bootstrap values were calculated as percentages of 1000 analyses, and values 50%–75% are indicated with a white circle, 76%–95% with a gray circle, and >96% with a solid black circle.

This review has provided the opportunity to re-examine some of these amplicon sequencing data using the MiDAS 2.1 database, to look at the distribution of *Chloroflexi* filamentous bacteria across multiple treatment plant settings in different countries (McIlroy et al., 2017b). To achieve this, the MiDAS 2.1 taxonomy and sequence database files were amended to include the newly named “*Ca*. Catenibacter,” “*Ca*. Amarithrix,” “*Ca*. Trichobacter,” and “*Ca*. Defluviithrix” described here (Supplementary Table S1). This exercise has resulted in the group originally representing “*Ca*. Defluviifilum” (previously MiDAS 1.2 group P2CN44) being divided into three clades each representing “*Ca*. Defluviifilum,” “*Ca*. Amarithrix,” and “*Ca*. Defluviithrix” (Figure 1). This decision was taken because of the propensity of their 16S rRNA sequences to fall into three distinct clades after phylogenetic analysis (Figures 1, 2), where members of each clade generally shared c.a. > 95% 16S rRNA gene sequence similarity (based on a shared 1070 bp 16S rRNA gene alignment), and their reported morphological and physiological differences (Yoon et al., 2010; Kragelund et al., 2011; Speirs et al., 2017; Nierychlo et al., 2019). We believe it is prudent at this time to separate these phylotypes, as there is no convincing evidence to suggest they are representatives of the same genus.

Data chosen for these analyses were based on the availability of high quality sequence data and PCR primer details, which had been generated with the Illumina platforms. The survey involved analyses of 288 individual *Chloroflexi* populations from eight countries in Asia, Europe and North America (Supplementary Data File S1). These data were analyzed using QIIME2 (Bolyen et al., 2018), and protocol details are given as Supplementary Information (Supplementary Data File S2). It was not within the capability of this review to assess which operational, environmental or geographical factors play any part in the distribution of these filaments, as in most cases this information was not provided. However, trends were seen in their distributions in plants both within and between countries, which warrant further investigation. This restricted survey serves as a means to reveal the global distribution and population abundances of these activated sludge *Chloroflexi*, using the updated version of the MiDAS 2.1 taxonomy database (McIlroy et al., 2017b).

Briefly, the data presented here (Table 4 and Supplementary Data File S1) reveal that “*Ca*. Villigracilis” and “*Ca*. Defluviithrix” sequences are widely distributed globally, being detected respectively in 81.3 and 71.2% of all the biomass samples examined. This is despite no mention of either of these in the identification manuals of Eikelboom and Jenkins (see earlier), and neither showed similarities to any of the morphotypes described there (Yoon et al., 2010; Nierychlo et al., 2019). Consequently all surveys based on microscopy and information in these manuals (Rossetti et al., 2017) would miss these. The filaments “*Ca*. Promineofilum,” “*Ca*. Sarcinithrix,” “*Ca*. Catenibacter,” and “*Ca*. Kouleothrix” were only slightly less frequently seen, occurring in 69%, 69%, 68% and 59% of biomass samples respectively, (Table 4 and Supplementary Data File S1).

**TABLE 4:** Summary of abundances of the activated sludge filamentous *Chloroflexi* phylotypes distributed across the amplicon data survey.

**Phylotype**	**Number of samples where phylotype had a relative abundance ≥0.1 (%)**	**Number of samples where phylotype was detected (%)**	**Mean relative abundance (%)**	**Median relative abundance (%)**	**Maximum relative abundance (%)**
“Ca. Villigracilis”	63.9	81.3	0.76	0.26	5.36
“*Ca*. Amarithrix”	19.4	36.5	0.19	0	1.94
“*Ca*. Defluviiflium”	2.1	6.3	0.54	0	5.33
“Ca. Defluviithrix”	45.1	71.2	0.44	0.09	4.25
“*Ca*. Catenibacter”	39.6	68.4	0.31	0.06	3.63
“Ca. Kouleothrix”	30.6	58.7	0.73	0.02	17.82
“Ca. Sarcinithrix”	52.4	69.4	0.50	0.13	5.06
“Ca. Amarolinea”	3.1	5.2	0.46	0	1.38
“*Ca*. Promineofilum”	58.7	68.8	1.07	0.22	9.51
“*Ca*. Trichobacter”	0	0	0	0	0

*For the complete data see Supplementary Data File S1.*

Conversely, despite it being a highly abundant filament in some samples from Danish plants (Andersen et al., 2019; Nierychlo et al., 2019), “*Ca*. Amarolinea” was detected sporadically in only 5.2% of the global samples examined here, although only those taken from Danish plants at regular intervals during the yearly sampling period to reveal any marked seasonal changes were analyzed. “*Ca* Trichobacter” was not detected at all (Table 4 and Supplementary Data File S1). However, these latter two low values may reflect their low representation in the taxonomic database, being limited to two reference sequences each. The average relative abundance of *Chloroflexi* filaments was usually low at approx. 0.5% of the total population, thus reflecting abundances described in earlier reports. More details on the occurrence of each filament are given below.

## Whole Genome Sequencing and Metagenomics of *Chloroflexi* in Activated Sludge

Activated sludge bacterial whole genome sequencing of pure cultures and metagenomic analyses of mixed communities containing organisms of interest have also been facilitated substantially by DNA ngs protocols, but have not yet been applied widely to the Eikelboom filament morphotypes. With uncultured filaments, analyses of enriched mixed cultures are commonly used to generate metagenomic data. The whole genome sequences of cultured and uncultured bulking filaments have been published (e.g., Lapidus et al., 2011; Kristiansen et al., 2013; McIlroy et al., 2013, 2015; Guo et al., 2015; Sekiguchi et al., 2015). These include the uncultured *Chloroflexi* filament “*Ca*. P. breve.” from a plant in Slovenia, and metabolic models have been constructed from it (McIlroy et al., 2016). The whole genome sequence of “*Ca*. Kouleothrix sp.” is also available (Ward et al., 2018), although incompletely annotated, as is that of “*Ca*. A. aalborgensis” (Andersen et al., 2019). Their major features will be discussed later. The value of having such information is that they reveal the complete potential metabolic road map of the organism, unlike MAR for example, where only a small snapshot of its physiology/metabolism is exposed. However, as McIlroy et al. (2018) emphasize, while genomes of most activated sludge bacteria of interest contain genes encoding proteins with the same or similar function, MAR is needed to demonstrate *in situ* whether these genes are expressed under the imposed plant conditions. Such metagenomic studies also provide the basis for transcriptomic studies looking at gene expression *in situ* in activated sludge populations e.g. (He and McMahon, 2011), but none have been published so far for *Chloroflexi* filaments.

## The *In Situ* Physiology of the *Chloroflexi* in Activated Sludge

Attempts have been made to understand the *in situ* cophysiology of the *Chloroflexi* in a wide range of habitats, including the different operating conditions encountered in activated sludge treatment processes designed to remove N and P. Early MAR studies with the bulking filaments have been summarized by Nielsen et al. (2009b). Often the only FISH probes used to identify these were the CFX1223/GNSB941 probes, and so little information was generated about the ecophysiology of individual *Chloroflexi* filament populations. The still limited data available would suggest the *Chloroflexi* are specialized feeders with very similar metabolic needs, which is surprising in a group so phylogenetically and ecologically diverse (Nierychlo et al., 2019). Data from several FISH/MAR studies generally agree that they have a clear preference especially for simple sugars like sucrose, and for complex polymers and their products and amino acids, not just in activated sludge processes, but also in Anammox and membrane bioreactors (Kragelund et al., 2007a; Miura et al., 2007; Kindaichi et al., 2012). Nierychlo et al. (2019) also suggested that glycerol and long chain fatty acids could be used by some. Genomic information from anaerobic and aerobic *Chloroflexi* characterized from sediments (Hug et al., 2013) and anaerobic bioreactors (McIlroy et al., 2017a; Petriglieri et al., 2018) suggested similar nutritional preferences for the novel *Chloroflexi* detected there. Lysed bacterial cell wall debris in the form of the peptidoglycan monomer N-acetyl glucosamine is also commonly utilized (Nielsen et al., 2009b; Nierychlo et al., 2019).

Microautoradiography evidence from activated sludge filaments fails to support any widespread ability to utilize acetate, a major metabolite in nutrient removal plants, although Kragelund et al. (2007a) have evidence that “*Ca.* Kouleothrix” may. Furthermore, the cultured strain ETI described by Yoon et al. (2010) also grows on acetate as well as lactate and pyruvate as sole carbon sources. Generally these substrates were only assimilated under aerobic conditions, although genomic evidence for “*Ca*. P. breve” (McIlroy et al., 2016; Nierychlo et al., 2019), and MAR data for “*Ca*. Defluviifilum” (Kragelund et al., 2011) (see below), would suggest that statement does not apply to all the ‘aerobic’ filaments characterized in activated sludge (see later). Nierychlo et al. (2019) also showed by FISH/MAR that their four previously uncharacterized *Chloroflexi* in Danish plants assimilated some but not all substrates anaerobically. They shared a similar physiology in being chemoorganoheterotrophic facultatively anaerobic fermenters, using predominantly carbohydrates as substrates, and hence are similar essentially to “*Ca.* P. breve” (McIlroy et al., 2016). It is still unclear whether these can denitrify. All assimilated sugars under anoxic conditions with NO_2_/NO_3_ as terminal electron acceptors, but none could nitrify. Their individual attributes are discussed in more detail in Nierychlo et al. (2019) and below.

Methods used to monitor the synthesis *in situ* of exocellular depolymerase enzymes reveal how well suited these filaments are for degradation of complex polymers, although the assay systems used did not always reveal individual enzyme substrate specificities. Thus, Kragelund et al. (2007a) showed with “*Ca*. Kouleothrix” and FISH probes targeting this filament, that it produced a range of polysaccharide degrading enzymes including chitinases, glucuronidases, as well as proteases (Nielsen et al., 2009b). Interesting, their *in situ* MAR determined metabolic attributes did not always agree with those obtained with the corresponding pure cultures of this filaments (Kragelund et al., 2007a). Later, “*Ca.* Defluviifilum” (Kragelund et al., 2011) was shown also to synthesizes exocellular enzymes able to metabolize a range of macromolecules. Such attributes suggest an active role for them in the hydrolysis of particulate and colloidal material suspended in the mixed liquor (Nierychlo et al., 2019). They could also assimilate glucose under aerobic and anoxic conditions with nitrite or nitrate acting as electron acceptors, consistent with them behaving as denitrifying bacteria *in situ* (but see above). With so few *Chloroflexi* cultured and so few *in situ* FISH/MAR studies performed outside of Denmark, it seems probable that globally their ecophysiology will eventually reflect more closely their considerable phylogenetic diversity, as already suggested by Ward et al. (2018) for “*Ca*. Kouleothrix” and by Sorokin et al. (2014) for the chemolithoautotrophic nitrifying non-filamentous *Nitrolancea hollandica*.

## Description of the *Chloroflexi* Present in Activated Sludge Communities

Sufficient FISH and 16S rRNA amplicon sequence data are now available to make the task of identifying novel members of activated sludge *Chloroflexi* more straightforward. Thus, retrieved cloned 16S rRNA sequences, in combination with appropriate FISH probe analyses allow probed plant samples to be screened, revealing which of the positive and negative probe target sites the clones of interest may possess (Speirs et al., 2009). Then new FISH probes can be designed against these clone sequences, and applied to samples, where only the filaments of interest will fluoresce. This approach has been used successfully to identify “*Ca*. Promineofilum,” “*Ca.* Defuviifilum,” “*Ca.* Sarcinithrix,” and “*Ca.* Trichobacter” (Speirs et al., 2009, 2011, 2015; Kragelund et al., 2011; McIlroy et al., 2016; Table 1). “*Ca*. Amarithrix” and “*Ca*. Catenibacter” were identified by a similar method, but using amplicon sequencing to retrieve the 16S rRNA gene sequences of interest instead (Speirs et al., 2017). Correspondingly, amplicon sequence data have been used to help identify and map the distribution of *Chloroflexi* in plants of different configurations in several countries, and in combination with new FISH probes designed against these sequences. Used with MAR, these have contributed toward the elucidation of the ecophysiology of novel *Chloroflexi* in Danish treatment plants (Andersen et al., 2019; Nierychlo et al., 2019).

The features of interest of individual *Chloroflexi* filaments detected initially by the phylum level FISH probes CFX1223 and GNSB941 in activated sludge plant samples, and from amplicon sequencing data are described next. Several schemes are available to classify and identify 16S rRNA gene sequences. These are all ‘special purpose’ systems based on different classificatory principles for individual user groups (Seviour, 2010b; Balvočiūtė and Huson, 2017), and often differ in how they nominate and name individual *Chloroflexi* clades (see McIlroy et al., 2015, 2017a). Hence, the classifications of their 16S rRNA sequences have in some cases changed as later systems have become available (see earlier, Table 3). For example, the “*Ca* Sarcinithrix” morphotype 0914 *Chloroflexi* filaments (Speirs et al., 2011) were considered initially to belong to the subgroup 1 of the *Chloroflexi*, a clade thought to embrace predominantly sequences derived from activated sludge (Björnsson et al., 2002). Later, these filaments were classified as members of the *Chloroflexi* class *Caldilineae*, using the quality checked and manually curated SILVA taxonomy (Quast et al., 2013; Speirs et al., 2015), and then lastly attributed to the *Chloroflexi* group ‘SJA-15’ with the more recent MiDAS taxonomies (McIlroy et al., 2015, 2017b). The MiDAS scheme is based on the SILVA taxonomy, but differs in that it has been curated to include more attributes pertaining to activated sludge organisms, and thus for use specifically by those investigating activated sludge microbial communities (McIlroy et al., 2015). Hence, the MiDAS taxonomy has become clearly the best option for identifying the filamentous *Chloroflexi* discussed here, and this taxonomy and nomenclature, where appropriate, has been used in the following section.

### Anaerolineae

**“*Ca.* Villigracilis”** (Nierychlo et al., 2019).

***Vi lli gra ci lis;* L. m. n. *Villo*, tuft of hair; L. fem. Adj. *gracilis*, slender slim.**

***Villigracilis*, a filamentous bacterium often occurring in bundles.**

“*Ca.* Villigracilis” was described by Nierychlo et al. (2019) as one of the more abundant and frequently seen *Chloroflexi* filamentous bacteria in Danish wastewater treatment plants, and plants elsewhere (Table 4 and Supplementary Data File S1). This filament was formerly referred to as phylotype ‘SBR1029’ in the MiDAS 1.2 release. It possesses thin (0.3–0.4 μm × 15–50 μm) filaments, which were always located within the flocs, where they probably contribute to the matrix supporting floc formation. They frequently grow as bundles. It was not possible to determine by FISH which of the Eikelboom filaments it most closely resembled because of its location (Björnsson et al., 2002; Nierychlo et al., 2019). “*Ca*. Villigracilis” is a member of the *Anaerolineaceae*, in the *Anaerolineae*, and is the only facultatively aerobic member described so far (Nierychlo et al., 2019), with the remainder being strictly anaerobic (Sekiguchi et al., 2001; Yamada et al., 2006, 2007; Sun et al., 2016; McIlroy et al., 2017a). Two subgroups, A and B are recognized, and probes CFX763A and CFX763B designed to target respectively each (Table 1) collectively cover > 60% of the MiDAS 2.1 database sequences for members of this genus (Nierychlo et al., 2019). The amplicon survey performed here support the view that this phylotype is a core activated sludge member, and is present in 81% of the samples analyzed from around the world (Table 4 and Supplementary Data File S1). The mean and maximum relative abundances were found to be 0.76 and 5.36% of the total population respectively in the plants examined here.

### Ardenticatenia

**“*Ca.* Promineofilum”** (McIlroy et al., 2016).

***Pro mi nee oh L. v. combining form of prominere, to project, to jut out; filum L. n. n. filament. Promineofilum, a short filament protruding from flocs.***

“*Ca.* Promineofilum” is the genus name proposed for the Eikelboom filament morphotype type 0092 (McIlroy et al., 2016). This filament was formerly referred to as phylotype ‘B45’ in the MiDAS 1.2 release. The morphotype appears as blunt ended rods protruding from the floc surface, or less commonly, loose in the bulk liquid. It has been associated with foaming, but more likely ends up there accidentally (de los Reyes, 2010), since its cells are not hydrophobic (McIlroy et al., 2016). The filaments stain a distinctive lilac color with the Neisser stain, and so can be recognized readily microscopically. Two Neisser positive morphological variants differing in their trichome diameters were seen by Speirs et al. (2009) in an EBPR plant in Australia, and the thicker variant is the one described here and by McIlroy et al. (2016). These filaments are seen in most activated sludge plants around the world (Table 4 and Supplementary Data File S1), but probably more frequently and at higher abundances in EBPR plants designed to remove N and P microbiologically, where the thicker variant is more abundant than its thinner relative, at least in Australia (Speirs et al., 2009).

McIlroy et al. (2016) have shown from genomic annotation of the Cfx-K genome of “*Ca*. P. breve” that this filament possesses two chromosomes and a plasmid. Its phylogeny is confused. Based on 16S rRNA sequence data, it is most closely related to the *Anaerolineae* or *Ardenticatenia*, and probably represents at least a novel order. The MiDAS 2.1 taxonomy places it currently within the *Ardenticatenia*. The *Candidatus* name “*Ca*. Promineofilum breve” has been proposed for the species from which the Cfx-K genome was derived (McIlroy et al., 2016). Parks et al. (2018) in their GTDB taxonomy have suggested it should be a founder member of a new order, the *Promineofilales.*

“*Ca.* P. breve” is a facultatively anaerobic chemoorganoheterotroph, and thus able to obtain energy by both aerobic respiration and fermentation, probably of carbohydrates. MAR experiments showed it could only assimilate glucose, and not amino acids or short chain fatty acids. Glycogen appears to be stored, allowing the filament to survive the inevitable periods of substrate limitation encountered in activated sludge systems. This ability to respire and ferment sugars may explain its increased competitiveness in EBPR removal plants (Jenkins et al., 2003), where it is able to grow under the anaerobic conditions encountered there. In conventional aerobic plants “*Ca*. Promineofilum” is seen less frequently, possibly because it is unable to compete with the obligatory aerobic members of the community.

Whether it can denitrify is still unclear. No putative nitrate reductase or nitric oxidase encoding genes were present, although a periplasmic nitrous oxide reductase was seen, which suggests nitrous oxide could act potentially as terminal electron acceptor in denitrification. Some genomic data suggest an ability to grow chemolithoautotrophically, possessing as it does the genes for CO_2_ fixation using the Calvin-Benson-Bassam cycle. However, no genes were seen to suggest any ability to oxidize inorganic energy sources. Like “*Ca*. Trichobacter,” these filaments fail to hybridize with any of the EUB338mix FISH probes (Table 2). Speirs et al. (2009) have designed two FISH probes, the CFX197 probe which hybridizes with the thicker 0092 morphotype while the CFX223 probe targeted the thinner variant (Table 1 and Figure 2). Whether these represent different ‘species’ or strains of this filament is unknown. The fluorescence image generated from FISH probing suggests the ribosomes are not distributed evenly in the cells, but in patches (Speirs et al., 2009). It is not clear why, but they may be attached to intracellular membranous structures like those reported for the *Chloroflexi* filaments described by Yoon et al. (2010).

Results from the amplicon data survey (Table 4 and Supplementary Data File S1) agree with and extend earlier observations, and indicate this filament is a common member of activated sludge communities, being present in 68.8% of biomsass samples examined where its mean relative abundance was 1.07% of the total population, and it attained a maximum relative abundance of 9.51%.

**“*Ca.* Trichobacter” (name proposed in this review; Supplementary Table S2).**

***Trichobacter*; *Tri ko bac ter.* Gr. neut. n. *tricho* hair; Gr. n. masc. *bac ter* rod. *Trichobacter*, a filamentous bacterium consisting of chains of rod shaped cells.**

“*Ca*. Trichobacter” is the name proposed here for the Eikelboom type 0803 morphotype filaments detected in Australian plants, which do not respond to the FISH probes designed by Kragelund et al. (2011) against “*Ca.* Defluviifilum,” which possesses the same morphotype. It is usually seen at low levels in Australian plants, with abundances well below those of “*Ca.* Defluviifilum” (Speirs et al., 2015).

The biomass sample from which it was isolated (Speirs et al., 2015) came from a domestic treatment plant in Victoria, Australia, where this filament was at very high abundance, and was held responsible for the bulking event occurring there. It belongs to the *Ardenticatenia*, and thus is phylogenetically distant to “*Ca.* Defluviifilum,” being more closely related to “*Ca*. Promineofilum” (Speirs et al., 2009; McIlroy et al., 2016), with which it shares the same EUB338mix probe variant target site mismatches (see above).

One probe is available for “*Ca.* Trichobacter” (Table 1 and Figure 2; Speirs et al., 2015). Seven of the 32 Australian activated sludge samples surveyed contained this filament. “*Ca*. Trichobacter” sequences were included in the amended MiDAS 2.1 database used here for the global amplicon data survey performed for this review (Table 4 and Supplementary Data File S1). However, no examples of this filament were detected in any other plant.

### Caldilineae

**“*Ca.* Defluviifilum”** (Nierychlo et al., 2019).

***De flu v’ i’ i’ fi’um.* L. n. *defluvium*, sewage; L. neut. N. filum, thread. *Defluviifilum*, a filamentous bacterium in sewage.**

“*Ca.* Defluviifilum” formerly referred to as the phylotype ‘P2CN44’ in the MiDAS 1.2 release (McIlroy et al., 2015), is the name proposed for the uncultured Eikelboom type 0803 filament described by Kragelund et al. (2011) in plants in Denmark, treating domestic and pulp and paper wastes. Phylogenetic analysis places it within the class *Caldilineae*. This filament possesses the morphological features considered diagnostic for this morphotype (Jenkins et al., 2003; Eikelboom et al., 2006), although being so few in number makes its morphotype microscopic ‘identification’ problematic (Kragelund et al., 2011). The short, straight hydrophobic filaments consisting of square/rectangular cells are often located on the floc surface, but may also form interfloc bridges. No PHA or polyP inclusions were revealed after staining, and no S^0^ granules detectable by phase contrast. It can be difficult to distinguish this filament from “*Ca*. Sarcinithrix” (see later), except that its filaments are thinner, and those in bundles tend to align in parallel, while those in “*Ca*. Sarcinithrix” often traverse each other. The FISH probes now available should resolve this problem.

FISH based surveys of Danish nutrient removal plants (Kragelund et al., 2011) reveal this filament is a common component of the community, and its relative abundances remained quite stable over time. FISH analyses showed it was always filamentous *in situ*, and “*Ca*. Promineofilum” filaments were always present with it. “*Ca*. Defluviifilum” has also been seen at consistently high abundance in an A2O EBPR plant in Japan over a 12 month period.

It was recommended (Kragelund et al., 2011) that their T-0803-0654 FISH probe be used to detect “*Ca*. Defluviifilum” in plants treating domestic wastes, while the T-0803ind-0642 probe was recommended for those treating industrial wastes. However, Speirs et al. (2015, 2017) have shown that the T0803-0654 probe target site also occurs in several other 16S rRNA sequences with as little as 73% sequence similarity to the “*Ca.* Defluviifilum” target group. Because of this, the abundance values for this phylotype have probably been artificially inflated in the data derived from FISH surveys (see above). Thus, we recommend that the T0803-0654 probe be no longer used and the T0803ind-0642 probe be used instead, since it was designed to be more specific (Table 1 and Figure 1).

As mentioned already, it was decided for this review to incorporate the “*Ca*. Amarithrix” and “*Ca*. Defluviithrix” phylotypes into the MiDAS 2.1 database (Figure 1), which resulted in the number of sequences attributed to the “*Ca*. Defluviifilum” decreasing substantially. The survey of amplicon data performed here (Table 4 and Supplementary Data File S1) indicate that “*Ca*. Defluviifilum” was present in 6% of activated sludge samples, with an average relative abundance of 0.57% of the total population, and a maximum relative abundance of 5.33%. These values are generally lower than those given in previous publications (e.g., McIlroy et al., 2015, 2017a; Nierychlo et al., 2019), although not unexpected, because of our reallocation of reference sequences representing this group. This strategy makes any further comparison with previous data problematic, and additional analyses will be required.

**“*Ca.* Defluviithrix” (name proposed in this review; Supplementary Table S2).**

***De fluv’ i’ i thrix* L. neut. n. *defluvium* sewage; Gr. fem.n. *thrix* hair; *Defluviithrix* a filamentous bacterium in sewage.**

In 2010, Yoon et al. (2010) cultured seven strains of an unbranched, aerobic, mesophilic filamentous *Chloroflexi* isolate ET1 in Korea, which they claimed was the first aerobic *Caldilineae* retrieved from activated sludge (Nierychlo et al., 2019). No apparent sheath was present, and septa were rarely seen. They were unable to relate it definitively to any of the Eikelboom filament morphotypes, but suggested it most closely resembled morphologically *Haliscomenobacter hydrossis*, a member of the *Bacteroidetes* (Seviour and Nielsen, 2010). Its 16S rRNA sequence is most closely related (92%) to that of “*Ca.* Defluviifilum” (described above) and “*Ca.* Amarithrix” (described below) (Figure 1). Unusual intracellular membrane arrangements and inclusion bodies of an unknown composition were seen. Evidence was presented suggesting that pure cultures of “*Ca*. Defluviithrix” strain ETI fitted the common *Chloroflexi* nutritional profiles, in being chemoorganoheterotrophic, and capable of using a limited range of biopolymers, organic acids and simple sugars (not glucose), but only under aerobic conditions (Yoon et al., 2010). No phylotype specific probes were reported for this filament. However, the CFX1A331 probe (Table 1), designed to target members of the *Caldilineae* including “*Ca*. Defluviithrix,” showed this filament was most often located within the flocs (Yoon et al., 2010).

“*Ca.* Defluviithrix” is widely distributed globally, being found in many plants in other parts of the world, as revealed from amplicon sequencing data where it occurred in 71% of the samples surveyed, at a mean relative abundance of 0.44% of the total population and maximum relative abundance of 4.25% (Table 4 and Supplementary Data File S1). As this phylotype is not included in the MiDAS database, comparisons with previous data are not possible, although it seems likely that “*Ca*. Defluviithrix” has contributed a substantial portion of the “*Ca*. Defluviifilum” sequences detected in previous amplicon studies. Indeed, here it was the most widely distributed member of the MiDAS 1.2 ‘P2CN44’ group that also includes “*Ca*. Defluviifilum” and “*Ca*. Amarithrix.” No success has been reported in culturing any bulking *Chloroflexi* filaments other than this strain and “*Ca*. Kouleothrix spp.,” although several anaerobic *Anaerolineae* filaments from a range of habitats other than activated sludge have been obtained in pure culture (Sekiguchi et al., 2003; Yamada et al., 2006, 2007; Sun et al., 2016; McIlroy et al., 2017a).

**“*Ca.* Amarithrix” (name proposed in this review; Supplementary Table S2).**

***A mar i thrix*; Gr. n. *a mar a*, sewage duct; N L. masc. n. thrix, thread; Gr. *Amarithrix* a filamentous bacterium from sewage.**

“*Ca.* Amarithrix” is the name proposed here for a filament sharing features with the Eikelboom morphotype 0675. In many microscopic surveys, this morphotype is often grouped with the Eikelboom type 0041 filament, because of claims (Seviour et al., 1990), that while both have characteristic abundant attached growth on their trichomes, consisting of almost square cells, they could not be separated reliably on the Eikelboom key attribute of differences in their trichome diameters. Type 0675 is described (Jenkins et al., 2003; Eikelboom et al., 2006) as being thinner than type 0041. Whether they represent different morphotypes of the same filament and hence share similar ecologies, or were two phylogenetically distinct filaments has been resolved for Australian strains. It is now clear that although both are members of the *Caldilineae* in the phylum *Chloroflexi* they represent two distinct phylotypes belonging to related taxa (Speirs et al., 2017; Figure 2). The CFXmix FISH probes are recommended for initial probing to eliminate filaments belonging to the *Betaproteobacteria*, followed by the Caldi-0678 probe for the *Caldilineae*, before the probes CFX194a and CFX194b designed by Speirs et al. (2017) are applied as a mix (Table 1), together with the helper and competitor probes designed for them. FISH based surveys with these two probes (Speirs et al., 2017) would suggest that “*Ca*. Amarithrix” is more commonly seen in nutrient removal than conventional plants in Australia. However, a few type 0675 morphotypes responded to the CFXmix probes, but not to the CFX194mix probes, suggesting that further phylogenetic diversity within the *Chloroflexi* exists for this morphotype. At this point no genome data have been generated for this organism. They fall into the MiDAS 2.1 defined “*Ca*. Defluviifilum” genus, but Speirs et al. (2017) suggest they should be placed currently into a separate genus, based on 16S rRNA sequence similarity values and morphological differences.

For the purposes of this review, we have amended the MiDAS 2.1 database to include “*Ca*. Amarithrix” (Supplementary Table S1), where it reallocates several “*Ca*. Defluviifilum” sequences (Figure 1). This filament was detected in 37% of samples with a mean relative abundance of 0.2% of the total populations and maximum relative abundance of 1.94% (Table 4 and Supplementary Data File S1).

**“*Ca.* Catenibacter” (name proposed in this review; Supplementary Table S2).**

***Ca ten i bac’ ter;* L. fem.n. Ca te n i chain; Gr. hyp. masc. *ba’cter* rod; *Catenibacter* a bacterium consisting of chain of rod shaped cells.**

“*Ca.* Catenibacter” is the name proposed here for the *Chloroflexi* Eikelboom morphotype type 0041. As mentioned above, Eikelboom (1975) separated these from type 0675 on differences in their trichome diameters, and type 0041 has been combined with type 0675 in most microscopy based surveys (Seviour et al., 1990; Kragelund et al., 2011). Both can now be separated and hence identified by FISH (Speirs et al., 2017). They share the same general morphological attributes and are distinguished by abundant attached growth on their trichomes. To date, neither has been cultured. FISH survey studies in the past have suggested that the 0041 morphotype is polyphyletic, since some have been claimed to respond to probes targeting members of the phylum “*Ca.* Saccharibacteria,” previously named TM7 (Hugenholtz et al., 2001; Thomsen et al., 2002; Müller et al., 2007; Mielczarek et al., 2012), and others with *Curvibacter* in the *Betaproteobacteria* (Thomsen et al., 2006).

However, the early surveys were performed before the FISH probes for the *Chloroflexi* (Björnsson et al., 2002) were available, and doubts have since been raised about the specificity of the TM7 FISH probes of Hugenholtz et al. (2001) against filamentous members of this phylum. Thus, Nittami et al. (2014) showed that none of the type 0041/0675 filaments they saw in either Australian or Japanese plants fluoresced with the TM7-305 probe designed to target the “*Ca*. Saccharibacteria” phylum, including putative type 0041 “*Ca*. Saccharibacteria” filaments (Hugenholtz et al., 2001). Instead their TM7 FISH probes hybridized consistently with “*Ca*. Kouleothrix” filaments responding positively to the CHL1851, EU25-1238 and CFXmix probes (see below).

Speirs et al. (2017) designed a FISH probe, which fluoresced only with filaments with the *Chloroflexi* type 0041 morphology (Table 1 and Figure 2). This CFX86 probe should be used together with the corresponding helper probes, which enhance fluorescence signal strength (Speirs et al., 2017). Surveys using this probe showed these filaments can attain very long trichome lengths (>1000 μm) and extend from flocs leading to interfloc bridging. It is widely distributed globally, being present in 69% of the samples analyzed (Table 4 and Supplementary Data File S1). Its mean relative abundance was similar to the other phylotypes at 0.31% of the total population and a maximum detected relative abundance of 3.63%. Its presence in plants did not always coincide with that of “*Ca.* Amarithrix.”

### Chloroflexi

**“*Ca.* Kouleothrix”** (Kohno et al., 2002).

The Eikelboom morphotype 1851 filaments often staining weakly Gram positive, are now considered to be members of the genus “*Ca*. Kouleothrix,” containing the filamentous isolate “*Ca*. K. aurantiaca” (Kohno et al., 2002; Kragelund et al., 2007a), and being only approx. 84% similar in their phylogeny to their nearest isolated relative, *Roseiflexus castenholtzii.* The Australian isolate shares little of this phenotype, being neither a gliding thermotolerant filament, nor one producing carotenoid pigments (Beer et al., 2002), yet it belongs to the phototrophic members in the class *Chloroflexia.* This observation confirms that close phylogenetic relatedness is not always reflected in a shared physiology/biochemistry.

The Japanese “*Ca*. Kouleothrix” cultured strains of Kohno et al. (2002) are 99% similar to the Danish cultured strain Ver9Iso2 (Kragelund et al., 2007a), but as both are only 93–95% similar to the Australian type 1851 cultured isolate of Beer et al. (2002), it seems likely that more than one species/genus exists. This filament is seen commonly in plants of all configurations treating both domestic and industrial wastes around the world (Beer et al., 2002; Kragelund et al., 2007a), and especially in Japan Nittami et al. (2017), where its excessive growth commonly leads to bulking. This often involves interfloc bridges of bundles of thin filaments (Beer et al., 2002; Nittami et al., 2017), which are usually, but not always, covered with attached epiphytic bacterial growth, where the attached cells appear distinctively perpendicular to the filament surface. The proclivity of this filament to cause bulking appears high, and analyses of 16S rRNA amplicon data (Table 4 and Supplementary Data File S1) show this filament was present in 59% of activated sludge samples. The average relative abundance was 0.7% of the total population, Real time qPCR methods (Nittami et al. (2017) have been applied to quantify “*Ca*. Kouleothrix” (type 1851) to determine if a threshold value existed for this filament above which bulking occurred, but comparisons with the FISH based data of Liao et al. (2004) are problematic. As well as using different quantitative methodologies, each used a different value for the contentious sludge volume index (Schuler and Jassby, 2007) to define what represents a bulking sludge.

The whole genome sequence of “*Ca*. K. aurantiaca” COM-B (JCM 19913) has revealed that it does not behave as a phototroph, despite it possessing the genes required for anoxygenic phototrophy (Ward et al., 2018). Thus, encoded are the complete bacteriochlorophyll biosynthetic pathway, a cytochrome bc complex and a Type 2 reaction center (RC2), as well as genes for RuBisCO and phosphoribulokinase. So it appears to be capable of CO_2_ fixation using the Calvin cycle pathway. However, missing are genes encoding for the 3-hydroxypropionate cycle, the pathway for CO_2_ fixation in the genera *Chloroflexus* and *Roseiflexus*, its closest relatives (Ward et al., 2018).

The probes CHL1851 and EU25-1238 have been used in surveys of European industrial wastewater treatment plants, where Kragelund et al. (2007a) showed that about 50% of EU-1238 positive “*Ca*. Kouleothrix” filaments failed to respond to the CHL1851 probe, even though the EU25 isolate possessed its target site. No explanation for this is available. In those plants treating largely domestic wastes, the CHL1851 probe could detect this filament morphotype. Thus, for the *in situ* detection of “*Ca*. Kouleothrix,” both these probes should be applied together (Table 1 and Figure 2). Guo and Zhang (2012) raised concerns about the apparent low specificity of the CHL1851 probe of Beer et al. (2002), and proposed a replacement FISH probe, T1851-2 for this filament. However, there are no reports of its use. They claimed then only few members of the *Chloroflexia* possessed the T-1851-2 probe probe target site, but suggested it was unsuitable for the type 1851 strain described by Kohno et al. (2002), having two mismatches with it.

**“SJA-15”**.

**“*Ca.* Sarcinithrix”** (Nierychlo et al., 2019).

“*Ca.* Sarcinithrix” is the name given by Nierychlo et al. (2019) to the Eikelboom morphotype 0914. It was first identified in a sample taken from a badly bulking sequencing batch reactor activated sludge plant treating domestic wastes in South Australia, operating with a long sludge age of 20 days (Speirs et al., 2011). It is a member of the MiDAS 2.1 class ‘SJA-15.’ The filaments respond to the EUB338 mix and CFX1223/GNSB941 mix probes. FISH based surveys suggested that “*Ca.* Sarcinithrix” was seen generally at higher relative abundances in nutrient removal plants operating at long sludge ages (Speirs et al., 2011) in Australia and Denmark (McIlroy et al., 2015). The two FISH probes CFX67a and CFX67b were the first designed against this type 0914 morphotype (Table 1 and Figure 2), and both hybridized to filamentous bacteria having the type 0914 morphology. Helper probes were essential for use with the general CFXmix and EUB338-1 probes, as well as for both the CFX67a and CFX67b probes, as discussed by Speirs et al. (2011). Similar requirements were reported by Nierychlo et al. (2019). As with “*Ca*. Promineofilum” (Speirs et al., 2009), an uneven FISH signal was seen from individual cells, suggesting uneven distributions of ribosomes in them (Speirs et al., 2011).

Nierychlo et al. (2019) showed subsequently, that the CFX67a probe did not impart fluorescence to the majority of “*Ca.* Sarcinithrix” filaments seen in Danish plants exposed to it, and failed to target several full-length sequences classified to this genus in the MiDAS databases (McIlroy et al., 2015, 2017b). They designed two new FISH probes CFX449 and CFX1151 (Table 1 and Figure 2), which they suggested should be used together, and which individually cover > 85% of the relevant sequences in the MiDAS 2.1 database. Helper probes were also designed, which failed to enhance fluorescence intensity, but generated a more even fluorescence signal (Nierychlo et al., 2019).

“*Ca*. Sarcinithrix” appears widespread globally and was detected in 69% of the biomass samples analyzed in the global survey (Table 4 and Supplementary Data File S1). Mean relative abundance in these samples was 0.50% of the total population, and a maximum relative abundance of 5.06%. As stated above, “*Ca*. Sarcinithrix” can cause serious bulking episodes (Speirs et al., 2011) and together with their widespread distribution, suggests this filament is an organism worthy of more attention.

**“*Ca.* Amarolinea”** (Andersen et al., 2019).

***A ma ro li’ ne. a* Gr. n. amaro conduit, channel, sewage duct; L.fem.n. linea, a thread, a line; Amarolinea a thread from a sewer.**

“*Ca.* Amarolinea,” previously referred to as ‘C10 SB1A’ in the MiDAS 1.2 database, replaces the earlier name “*Ca.* Amarilinum,” and belongs to the MiDAS 2.1 class ‘SJA-15,’ which also contains “*Ca.* Sarcinithrix,” and with which it shares several metabolic traits (Andersen et al., 2019). The only FISH probe available, CFX64 (Table 1), was designed against abundant amplicon OTUs 3 and 4592 detected in Danish activated sludge samples (Nierychlo et al., 2019) and the available most closely related full-length sequences. Nierychlo et al. (2019) claim this probe should cover most of the “*Ca.* Amarolinea” filaments in Danish plants, although how common this filament was elsewhere was not known then. The survey of amplicon data conducted here indicates its distribution is not widespread, being detected in only 5.2% of biomass samples examined here (Table 4 and Supplementary Data File S1). Its relative abundance in these samples was comparable to those of other *Chloroflexi* phylotypes at 0.5% of total cell community, with a maximum relative abundance of 1.4%. Andersen et al. (2019) have shown that relative abundances as high as 30% have been recorded in some EBPR plants in Denmark for this filament, but even in these plants, abundances were often much lower at other sampling times and in some cases undetectable. At high abundance it probably contributes to sludge bulking episodes (Andersen et al., 2019). Thus, few “*Ca*. Amarolinea” sequences are present in public 16S rRNA gene sequence databases, and a BLAST search of the Genbank database using the “*Ca*. Amarolinea” 16S rRNA sequence (MH537630) as reference, and including those generated by amplicon sequencing (approx. 100 - 500bp), revealed only 17 entries with greater than 95% similarity to it. Exceptions to this are those in MBR plants in Korea and especially Taiwan, where “*Ca.* Amarolinea” was at high abundance in the all the samples examined, which were taken in the winter (Table 4 and Supplementary Data File S1). It should also be mentioned here that Jiang et al. (2018) showed it was a highly abundant filament (18% of total 16S rRNA sequences) in a foaming anoxic reactor in Hong Kong, depite the fact that it does not behave as a hydrophobic filament *in situ* (Andersen et al., 2019).

It is a non-motile Gram-negative filament (1–2.2 μm × 20–140 μm) with rectangular cells and no visible septa, staining violet/blue with the Neisser stain, and lacking polyphosphate and polyhydroxyalkanoate storage granules. Based on the distinctive Neisser staining reaction, this filament most closely resembles the Eikelboom type 0092 morphotype, as it too is Neisser positive in being distinctively violet/blue. However, the short blunt-ended filaments are thicker (1.5 μm) than those seen with “*Ca*. Promineofilum,” and are usually located entirely within the flocs, making them difficult to see microscopically.

A genome has now been sequenced for a member of this genus, and has been given the name “*Ca*. Amarolinea aalborgensis” (Andersen et al., 2019). The phylogeny of this filament appears to differ depending on the assessment criteria. 16S rRNA gene sequence analysis indicates it is a member of the class ‘SJA-15’ (Figure 2), while protein analyses place it within the class *Anaerolineae*. Based on the GTDB database of single copy marker genes (Parks et al., 2018), Andersen et al. (2019) propose that it should now be placed in a new family within the order *Caldilineales*, the *Amaroliniaceae*, with “*Ca*. Amarolinea, aalborgensis” sp. nov. as its sole member.

It is facultatively anaerobic, being able to ferment carbohydrates as well as being capable of aerobic respiration with a fully functional tricarboxylic acid cycle and cytochrome C oxidase as terminal transport chain donor. All the genes needed for glycolysis are present. “*Ca* Amarolinea” can carry out dissimilatory NO_3_ reduction to NH_3__,_ possessing genes encoding nitrate and nitrite reduction, but lacking the genes for nitrite acid and nitc acid reductases. It can also store anaerobically glycogen, which may be used as a potential energy source (Andersen et al.,2019), and thus enable it to compete anaerobically with the PAO *Tetrasphaera* spp. (see earlier) in EBPR plants.

### Thermomicrobia

#### *Nostocoida limicola* II *Chloroflexi*

While the Eikelboom *Nostocoida limicola* II morphotype has been embraced within the *Alphaproteobacteria* and *Actinobacteria* (Seviour and Nielsen, 2010), it is also shared by a filamentous member of the *Chloroflexi*, class *Thermomicrobia* (Hugenholtz and Stackebrandt, 2004), and has been cultured (Schade et al., 2002).

The probe AHW183 (Schade et al., 2002) designed from the 16S rRNA sequences of four cultured isolates is the only probe available (Table 1 and Figure 2), although it has rarely been used in published surveys, and so little is known of the occurrence of this filament globally. Furthermore, the accession for this probe target (HM316086) is not listed in the MiDAS 2.1 database as representing a filamentous population, and its identification proceeds only to order level (‘JG30-KF-CM45’) within the class *Thermomicrobia.*

#### *Nitrolancea hollandicus* (Sorokin et al., 2014)

The isolation of a non-filamentous nitrite oxidizing nitrifying *Chloroflexi*, in the class *Thermomicrobia* from a lab-scale nitrifying bioreactor operating at 35°C by Sorokin et al. (2012, 2014) is a reminder of how poor our present understanding of activated sludge *Chloroflexi* diversity and ecophysiology currently is. FISH probes have also been described for its *in situ* identification (Table 1). These have shown that, as with the proteobacterial Nitroso and Nitro bacteria discussed earlier, *N. hollandica* occurs as large clusters located closely adjacent to the Nitroso bacteria, with whom they presumably form a mutualistic relationship similar to that already discussed above for the other nitrifying bacteria.

The key question is whether *N. hollandicus* is present and active in large-scale nitrifying activated sludge systems. Sorokin et al. (2012) suggest from its ecophysiology that this organism was unlikely to survive there because, being an ‘r-strategist,’ it requires high concentrations of ammonia and nitrite for energy production and growth. Its ability to grow between 25°C and 63°C, higher temperatures than those reached in European treatment plants at least, may also preclude its presence. Certainly FISH probes designed to target its 16S rRNA genes and qPCR protocols failed to detect it in activated sludge treatment plants in European plants, although a 16S rRNA sequence closely related to *N. hollandicus* was detected in a SHARON reactor running at higher temperatures in Korea (Sorokin et al., 2012).

So unexpectedly, the survey data generated in this study showed it was present on several occasions in four plants of those examined here, (data not shown) Randers, Lundtofte, Haderslev and Ouyang. The first three are EBPR plants in Denmark and the fourth is a N removal plant in China. This finding raises the prospect that activated sludge plants around the world may contain non-filamentous *Chloroflex*i with important defined functional roles, and requires further changes to the existing paradigm of the microbiology of nitrification.

It is the only known nitrite oxidizing bacterium, which is not a member of the *Proteobacteria*, and evidence presented by Sorokin et al. (2012) would suggest it did not evolve from them. *N. hollandicus* lacks any of the intracellular cytoplasmic membranes seen in the proteobacterial nitrifiers, and their distinctively different lipids. The similarity of its nitrite oxidoreductase encoding *nrx* complex to those in *Nitrobacter* and *Nitrococcus* suggests lateral gene transfer of this complex occurred between them.

## Conclusion

Application of the molecular methods discussed here and elsewhere have shown repeatedly that so far unidentified and uncultured *Chloroflexi* are commonly present in activated sludge plants, especially since the data available presently come from a few countries only. Consequently our understanding of their phylogenetic diversity is far from complete. Andersen et al. (2019) and Nierychlo et al. (2019) have made important contributions toward a better understanding of their presence in Danish activated sludge plants, and we believe this review begins to add a global perspective, and will encourage others to pursue this fascinating group of bacteria.

Problems encountered here were often in comparing phylogenetic data generated in different studies. Much of these arose from subjective choices of DNA extraction methods and of PCR primers, often targeting different variable regions of the 16S rRNA. It is recommended that all future studies should use a standardized protocol for both obtaining and handling DNA samples. The experimentally validated suggestions of Albertsen et al. (2015) provide a sound basis for this.

Recently published surveys of 16S rRNA sequence data generated using PCR based protocols from 269 plants around the world supported by the Global Water Microbiome Consortium (Wu et al., 2019) and the imminent release of survey data based on the full-length 16S rRNA gene sequencing method described by Karst et al. (2018) carried out by the Center for Microbial Communities (Aalborg, Denmark) will surely increase dramatically our understanding of the activated sludge microbiome, and raise important questions for the future. The *Chloroflexi* will figure strongly there.

So there is an opportunity now to consolidate *Chloroflexi* amplicon data from plants globally, and extend substantially the data presented in this review from smaller numbers of studies. It is hoped that the user- friendly MiDAS database will be expanded to include these new data sets. However, as such surveys do not embrace every known treatment plant, and examine small sample numbers from each plant, there is still a place for long term studies carried out on individual plants. The important role of MIDAS in the future will be devalued unless journals insist that all published amplicon sequence data be deposited in publically available databases, for future compilation and analyses. This does not always occur presently.

Ultimately, the availability of relative abundance and environmental data, combined with the physiological information demonstrated experimentally with MAR and/or inferred from genome sequences, will surely assist in providing clues needed to understand more fully their roles in activated sludge plants. In the near future transcriptomic studies exploiting the rapidly increased generation of whole genome sequence data will extend the limitations of the FISH/MAR approach, allowing elucidation of the dynamics of *in situ* gene expression of these important bacteria, and help in solving the operational problems they cause.

## Dedication

This manuscript is dedicated to the memory of Professor Valter Tandoi, who made an important contribution to our present understanding of the bulking filamentous bacteria in activated sludge processes.

## Author Contributions

RS and LS conceived the idea and its elements for this review. LS designed and undertook the amplicon, 16S rRNA sequence, and FISH probe analyses, and prepared the tables and figures with the assistance from DR and SP. All authors discussed and contributed to the preparation of the final manuscript.

## Conflict of Interest Statement

The authors declare that the research was conducted in the absence of any commercial or financial relationships that could be construed as a potential conflict of interest.

## Footnotes

1 www.midasfieldguide.com

## Supplementary Material

TABLE S1 Summary of updates to the MiDAS 2.1 database undertaken for the additional *Chloroflexi* filaments named and described in this review. The phylogenetic clustering these changes are based upon are those shown in Figure 1.

TABLE S2 Suggested descriptive nomenclature and characteristics of the newly proposed *Candidatus Chloroflexi* genera described in the review.

DATA FILE S1 Data for the amplicon data sets surveyed for this review. The relative abundance for each *Chloroflexi* filament phylotype is expressed as a percentage of the total number of 16S rRNA gene sequences from each sample remaining after quality control. Analyses were undertaken using QIIME 2 (release 2017.12). Samples retaining fewer than 4000 features after quality control are marked by red text (total features column), since the detection threshold fell below 0.05% relative abundance. Relative abundance values are given and also shaded by their respective heatmap color.

DATA FILE S2 Step-wise protocol used for retrieving relative abundances of *Chloroflexi* from published databases of NGS 16S rRNA amplicon sequence data presented Supplementary Data File S1.
