# Supplementary material for: The Phylogeny, Biodiversity, and Ecology of the Chloroflexi in Activated Sludge
Source: Front Microbiol. 2019 Sep 13;10:2015. doi: 10.3389/fmicb.2019.02015 (PMC6753630; doi:10.3389/fmicb.2019.02015)
Supplement: TABLE S1 — Summary of updates to the MiDAS 2.1 database undertaken for the additional Chloroflexi filaments named and described in this review. The phylogenetic clustering these changes are based upon are those shown in Figure 1. [file Table_3.docx]

**Supplementary Table S1:** Summary of updates to the MiDAS 2.1 database undertaken for the additional *Chloroflexi* filaments named and described in this review. The phylogenetic clustering these changes are based upon are those shown in Figure 1.

| **Accession** | **Original Phylotype** | **Original taxonomy string** | | **Updated Phylotype** | **New Taxonomy string** |
| --- | --- | --- | --- | --- | --- |
| HQ262531 | “*Ca*. Defluviifilum” | k__Bacteria;p__Chloroflexi;c__Caldilineae;o__Caldilineles;f__Caldilineaceae;g__Candidatus Defluviifilum;s__ | | “*Ca*. Defluviifilum” | k__Bacteria;p__Chloroflexi;c__Caldilineae;o__Caldilineles;f__Caldilineaceae;g__Candidatus Defluviifilum;s__ |
| HQ014653 | “ | “ | | “ | “ |
| JQ180412 | ” | “ | | “ | “ |
| EU875524 | “ | “ | | “*Ca*. Defluviithrix” | k__Bacteria;p__Chloroflexi;c__Caldilineae;o__Caldilineles;f__Caldilineaceae;g__Candidatus ET1;s__ |
| AB630554 | “ | “ | | “ | “ |
| FN824844 | “ | “ | | “ | “ |
| FQ659939 | “ | “ | | “ | “ |
| AJ504591 | “ | “ | | “ | “ |
| EU134006 | “ | “ | | “ | “ |
| CU922033 | “ | “ | | “ | “ |
| JX079302 | “ | “ | | “ | “ |
| FJ542929 | “ | “ | | “ | “ |
| KC683035 | “ | “ | | “ | “ |
| KC683244 | “ | “ | | “ | “ |
| KP204493 | “ | “ | | “*Ca*. Amarithrix” | k__Bacteria;p__Chloroflexi;c__Caldilineae;o__Caldilineles;f__Caldilineaceae;g__Candidatus Amarithrix;s__ |
| JN391831 | “ | “ | | “ | “ |
| KF697457 | “ | “ | | “ | “ |
| KF697498 | “ | “ | | “ | “ |
| CU918207 | “ | “ | | “ | “ |
| CU921890 | “ | “ | | “ | “ |
| CU926187 | “ | “ | | “ | “ |
| AB354620 | “uncultured” | k__Bacteria;p__Chloroflexi;c__Caldilineae;o__Caldilineles;f__Caldilineaceae;g__uncultured;s__ | | “*Ca*. Catenibacter” | k__Bacteria;p__Chloroflexi;c__Caldilineae;o__Caldilineles;f__  Caldilineaceae;g__Candidatus Catenibacter;s__ |
| AB630545 | “ | “ | | “ | “ |
| HQ343217 | “ | “ | | “ | “ |
| JN178510 | “ | “ | | “ | “ |
| AY491567 | “ | “ | | “ | “ |
| HQ828001 | “ | “ | | “ | “ |
| JN869072 | “ | “ | | “ | “ |
| AB630564 | “ | “ | | “ | “ |
| HQ697512 | “ | “ | | “ | “ |
| HQ697529 | “ | “ | | “ | “ |
| KJ548904 | “ | “ | | “ | “ |
| HQ014651 | “ | “ | | “ | “ |
| **New additions to the MiDAS 2.1 database** | | | | | |
| KP835206 | n/a | NA | “*Ca*. Trichobacter” | | k__Bacteria;p__Chloroflexi;c__Ardenticatenia;o__419;f__2-1;g__Candidatus Trichobacter;s__ |
| KP835207 | n/a | NA | “ | | “ |

NA = Not Applicable.

**Supplementary Table S2:** Suggested descriptive nomenclature and characteristics of the newly proposed *Candidatus* *Chloroflexi* genera described in the review.

| **Attribute** | **Specifications** |
| --- | --- |
| Status | *Candidatus* |
| Vernacular epithet | ‘another’ |
| Phylogenetic lineage or possible genus | Genus Trichobacter in the *,Chloroflexi, Ardenticatenia* |
| Cultivation | Not cultivated |
| Gram reaction | Gram negative |
| Morphology | Filamentous |
| Basis of assignment | 16S rRNA gene sequence (KP835206, KP835207) |
| Specified identification of morphotype | Oligonucleotide sequences complementary to unique region of 16S rRNA: 5’- CAGATCACTACCACCGTC -3’ (competitor 5’- CAGATCACTACCACCAGA-3’) |
| Habitat, association, or host | Activated sludge |
| Metabolism and unusual features | Filament with similar morphology to Eikelboom morphotype 0803 |
| Growth temperature | Mesophile |
| Source | Full-scale biological nutrient removal wastewater treatment plants in Australia |
| Author(s) | Speirs *et al*. (2015) |

| **Attribute** | **Specifications** |
| --- | --- |
| Status | *Candidatus* |
| Vernacular epithet | ‘another’ |
| Phylogenetic lineage or possible genus | Genus Defluviithrix in the *Chloroflex*i in the family *Caldiliniaceae* |
| Cultivation | Cultured |
| Gram reaction | Gram variable |
| Morphology | Filamentous |
| Basis of assignment | 16S rRNA gene sequence (EU875524) |
| Specified identification of morphotype | Oligonucleotide sequences complementary to  unique region of 16S rRNA: None provided |
| Habitat, association, or host | Activated sludge |
| Metabolism and unusual features | Aerobic chemoorganoheterotrophic filament. Not similar to any Eikelboom morphotype |
| Growth temperature | Mesophile |
| Source | Full-scale wastewater treatment plant in Korea |
| Author(s) | Yoon *et al*. (2010) |

| **Attribute** | **Specifications** |
| --- | --- |
| Status | *Candidatus* |
| Vernacular epithet | ‘another’ |
| Phylogenetic lineage or possible genus | Genus Amarithrix in the *Chloroflexi* in the family *Caldiliniaceae* |
| Cultivation | Not cultured |
| Gram reaction | Gram variable |
| Morphology | Filamentous, resembling Eikelboom morphotype 0675 |
| Basis of assignment | 16S rRNA gene sequence (HQ262528, HQ262533) |
| Specified identification of morphotype | Oligonucleotide sequences *complementary* to unique region of 16S rRNA:  5’- GCGCCAGAGCTTTCCCCA-3’, 5’-GCACCAGRGCTTTCCCCA-3’  (competitors: 5’-GCGCCAGAGCTTTCCCCC-3’, 5’-CCGCCAGAGCTTTCCCCA-3’)  (helpers: 5’-AACACCTTCCCAGGCGTCTGG-3’, 5’-CGGAYGCAGACCCCTCCYRRA-3’,  5’-CATCTCTTCCCAGAAATATGGATGTATG-3’) |
| Habitat, association, or host | Activated sludge |
| Metabolism and unusual features | Filament with similar morphology to Eikelboom morphotype 0675 |
| Growth temperature | Mesophile |
| Source | Full-scale biological nutrient removal wastewater treatment plant |
| Author(s) | Speirs *et al*. (2017) |

| **Attribute** | **Specifications** |
| --- | --- |
| Status | *Candidatus* |
| Vernacular epithet | ‘another’ |
| Phylogenetic lineage or possible genus | Genus Catenibacter in the Chloroflexi, in the family *Caldilniaceae* |
| Cultivation | Not cultured |
| Gram reaction | Gram variable |
| Morphology | Filamentous, Eikelboom morphotype 0041 |
| Basis of assignment | 16S rRNA gene sequence (JN391658) |
| Specified identification of morphotype | Oligonucleotide sequences complementary to unique region of 16S rRNA:  5’-CCGCCACTTTCARGGATA C-3’ (helpers: 5’- AWGTACCCYCTCACGTTCGAC -3’, 5’-WCCTACGTSTTACKCACCCGT-3’) |
| Habitat, association, or host | Activated sludge |
| Metabolism and unusual features | Filament with similar morphology to Eikelboom morphotype 0041 |
| Growth temperature |  |
| Source | Full-scale biological nutrient removal wastewater treatment plant |
| Author(s) | Speirs *et al*. (2017) |
