# Supplementary material for: The Phylogeny, Biodiversity, and Ecology of the Chloroflexi in Activated Sludge
Source: Front Microbiol. 2019 Sep 13;10:2015. doi: 10.3389/fmicb.2019.02015 (PMC6753630; doi:10.3389/fmicb.2019.02015)
Supplement: DATA FILE S2 — Step-wise protocol used for retrieving relative abundances of Chloroflexi from published databases of NGS 16S rRNA amplicon sequence data presented Supplementary Data File S1. [file Table_2.DOCX]

**Supplementary Data File S2:** QIIME2 commands for the analysis of NGS amplicon data. Commands given in grey text, user nominated file names and values given in blue (note and maintain the file formats). Sequence data was downloaded from either the NCBI Sequence Read Archive (SRA), European Nucleotide Archive (ENA) or DNA Data Bank or Japan (DDBJ). Sequencing primers and data accession numbers are available in the original publications, and listed in Supplementary Data File S1. Read trim lengths were subjectively determined when viewing the data (file file-name_demux.qzv).

**Files required:**

sequence data (.fastq)
manifest file (.txt, .tsv)
metadata file (.txt, .tsv)
MiDAS sequence file (.fasta)
MiDAS taxonomy file (.tax)

**QIIME2 commands:**

**1.** SINGLE-END data manifest file import

qiime tools import --type 'SampleData[SequencesWithQuality]' --input-path file-name --output-path file-name_demux.qza --source-format SingleEndFastqManifestPhred33

OR

PAIRED-END data manifest file import

qiime tools import --type 'SampleData[PairedEndSequencesWithQuality]' --input-path file-name --output-path file-name_demux.qza --source-format PairedEndFastqManifestPhred33

**2.** Viewing imported data (Note the appropriate values for trimming the data when viewing “file-name_demux.qzv”)

qiime demux summarize --i-data file-name_demux.qza --o-visualization file-name_demux.qzv

qiime tools view file-name_demux.qzv
  

**3.** SINGLE-END quality control/denoise

qiime dada2 denoise-single --i-demultiplexed-seqs file-name_demux.qza --o-representative-sequences file-name_rep_seqs.qza --o-table file-name_table.qza --p-trim-left VALUE --p-trunc-len VALUE

OR

PAIRED-END quality control/denoise

qiime dada2 denoise-paired --i-demultiplexed-seqs file-name_demux.qza --o-table file-name_table --o-representative-sequences file-name_rep_seqs.qza --p-trim-left-f VALUE --p-trim-left-r VALUE --p-trunc-len-f VALUE --p-trunc-len-r VALUE

**4.** Database (MiDAS) import and training

qiime tools import --type 'FeatureData[Sequence]' --input-path MiDAS_S123_2.1.3_Speirs_alt.fasta --output-path MiDAS_S123_2.1.3_Speirs_alt.qza

qiime tools import --type 'FeatureData[Taxonomy]' --source-format HeaderlessTSVTaxonomyFormat --input-path MiDAS_S123_2.1.3_Speirs_alt.tax --output-path MiDAS_S123_2.1.3_Speirs_alt_ref_taxonomy.qza

qiime feature-classifier extract-reads --i-sequences MiDAS_S123_2.1.3_Speirs_alt.qza --p-f-primer FORWARD PRIMER SEQUENCE --p-r-primer REVERSE PRIMER SEQUENCE --p-trunc-len VALUE --o-reads MiDAS_S123_2.1.3_Speirs_alt_Extract.qza

qiime feature-classifier fit-classifier-naive-bayes --i-reference-reads MiDAS_S123_2.1.3_Speirs_alt_Extract.qza --i-reference-taxonomy MiDAS_S123_2.1.3_Speirs_alt_ref_taxonomy.qza --o-classifier MiDAS_Speirs_alt_classifier.qza


**5.** Taxonomic analysis and presentation

qiime feature-classifier classify-sklearn --i-classifier MiDAS_Speirs_alt_classifier.qza --i-reads file-name_rep_seqs.qza --o-classification file-name_taxonomy.qza

qiime taxa barplot --i-table file-name_table.qza --i-taxonomy file-name_taxonomy.qza --m-metadata-file file-name_metadata.tsv --o-visualization file-name_taxa_bar_plots.qzv

qiime tools view file-name_taxa_bar_plots.qzv

**6.** Relative abundance values are then exported (from file-name_taxa_bar_plots.qzv) in CSV format and imported to Microsoft Excel.
